# Clinical phenotypes of Alzheimer’s disease: investigating atrophy patterns and their pathological correlates

**DOI:** 10.1186/s13195-025-01727-5

**Published:** 2025-04-26

**Authors:** Niels Reijner, I. Frigerio, M. M. A. Bouwman, B. D. C. Boon, N. Guizard, T. Jubault, J. J. M. Hoozemans, A. J. M. Rozemuller, F. H. Bouwman, F. Barkhof, E. Gordon, W. D. J. van de Berg, L. E. Jonkman

**Affiliations:** 1 Department of Anatomy and Neurosciences, Amsterdam UMC, Vrije Universiteit Amsterdam, De Boelelaan 1108, Amsterdam, 1081 HZ Netherlands; 2 Programs of Neurodegeneration, Amsterdam Neuroscience, Boelelaan 1117, Amsterdam, Netherlands; 3 Programs of Brain Imaging, Amsterdam Neuroscience, Boelelaan 1117, Amsterdam, Netherlands; 4 Department of Neuroscience, Mayo Clinic, 4500 San Pablo Road, Jacksonville, USA; 5 Qynapse, 2 - 10 Rue d’Oradour-Sur-Glane, Paris, 75015 France; 6 Department of Pathology, Amsterdam UMC, Vrije Universiteit Amsterdam, Amsterdam, Netherlands; 7 Department of Neurology, Amsterdam UMC, Vrije Universiteit Amsterdam, Amsterdam, Netherlands; 8 Department of Radiology and Nuclear Medicine, Amsterdam UMC, Vrije Universiteit Amsterdam, Amsterdam, Netherlands; 9 Institutes of Neurology and Healthcare Engineering, University College London, Gower Street, London, UK

**Keywords:** Alzheimer’s disease, Cortical volume, Clinical phenotypes, Pathological features, Post-mortem

## Abstract

**Background:**

In Alzheimer’s disease (AD), MRI atrophy patterns can distinguish between amnestic (typical) and non-amnestic (atypical) clinical phenotypes and are increasingly used for diagnosis and outcome measures in clinical trials. However, understanding how protein accumulation and other key features of neurodegeneration influence these imaging measurements, are lacking. The current study aimed to assess regional MRI patterns of cortical atrophy across clinical AD phenotypes, and their association with amyloid-beta (Aβ), phosphorylated tau (pTau), neuro-axonal degeneration and microvascular deterioration.

**Methods:**

Post-mortem *in-situ* 3DT1 3 T-MRI data was obtained from 33 AD (17 typical, 16 atypical) and 16 control brain donors. Additionally, ante-mortem 3DT1 3 T-MRI scans of brain donors were collected if available. Regional volumes were obtained from MRI scans using an atlas based parcellation software. Eight cortical brain regions were selected from formalin-fixed right hemispheres of brain donors and then immunostained for Aβ, pTau, neurofilament light, and collagen IV. Group comparisons and volume-pathology associations were analyzed using linear mixed models corrected for age, sex, post-mortem delay, and intracranial volume.

**Results:**

Compared to controls, both typical and atypical AD showed volume loss in the temporo-occipital cortex, while typical AD showed additional volume loss in the parietal cortex. Posterior cingulate volume was lower in typical AD compared to atypical AD (- 6.9%, *p* = 0.043). In AD, a global positive association between MRI cortical volume and Aβ load (βs = 0.21, *p* = 0.010), and a global negative association with NfL load (βs = - 0.18, *p* = 0.018) were observed. Regionally, higher superior parietal gyrus volume was associated with higher Aβ load in typical AD (βs = 0.47, *p* = 0.004), lower middle frontal gyrus volume associated with higher NfL load in atypical AD (βs = - 0.50, *p* < 0.001), and lower hippocampal volume associated with higher COLIV load in typical AD (βs = - 1.69, *p* < 0.001). Comparing post-mortem with ante-mortem scans showed minimal volume differences at scan-intervals within 2 years, highlighting the translational aspect of this study.

**Conclusion:**

For both clinical phenotypes, cortical volume is affected by Aβ and neuro-axonal damage, but in opposing directions. Differences in volume-pathology relationships between clinical phenotypes are region-specific. The findings of this study could improve the interpretation of MRI datasets in heterogenous AD cohorts, both in research and clinical settings.

**Supplementary Information:**

The online version contains supplementary material available at 10.1186/s13195-025-01727-5.

## Background

The hallmark of Alzheimer’s disease (AD) is neurodegeneration of cortical brain tissue, due to a cascade of amyloid-beta (Aβ) and phosphorylated tau (pTau) protein accumulation, leading to cortical atrophy. MRI measured atrophy shows to correlate with cognitive decline and could therefore be used as a diagnostic and monitoring tool in clinical settings as well as in research and clinical trials [1–3]. Atrophy patterns are heterogenous in the clinical AD spectrum, which can be observed in different clinically defined subtypes [4, 5]. The typical AD subtype, characterized by initial dominant amnestic deficits (i.e. memory impairment), primarily exhibits atrophy in cortical regions related to memory processing, such as the medial temporal regions and subsequent parietal regions [3]. In contrast, atypical AD subtypes, characterized by initially dominant non-amnestic cognitive symptoms including behavioral [6], dysexecutive [7], visuospatial [8] or logopenic [9] deficits, have shown to exhibit specific atrophy patterns in brain regions related to these cognitive deficits [4, 5]. For example, compared to typical AD, both the dysexecutive and behavioral subtypes show frontal atrophy, with the former exhibiting broader cortical involvement and the latter a more focal pattern [6, 7, 10]. Whereas the visuospatial subtype primarily shows a more occipital-parietal or occipito-temporal pattern [8]. One of the lesser understood aspects of these differential atrophy patterns is their relationship to the neuropathological features of AD. Previous research has focused primarily on the impact of hallmark features of AD, namely the accumulation of Aβ and pTau proteins, on subtype-specific atrophy patterns [11–13]. These studies suggest that the distribution patterns of pTau, rather than Aβ, play a key role in differentiating AD subtypes, and are strongly associated with atrophy patterns. In addition to the pathological protein accumulations, additional neurodegenerative features likely contribute to subtype-specific atrophy patterns in AD, such as neuro-axonal degeneration and microvascular deterioration.

One key non-protein accumulating pathological feature in AD is neuro-axonal degeneration [14, 15]. A decrease in axonal function impairs cellular transport and signal transduction, leading to neurodegeneration [16–18]. Neuro-axonal degeneration has been especially well characterized by studies focusing on the neurocytoskeletal protein marker neurofilament light (NfL) [19–21]. Primarily studied in CSF and plasma, NfL levels have been shown to increase with higher atrophy rates in AD [22, 23]. A study by Paterson, Toombs [24] showed different levels of CSF NfL between typical and atypical AD, and its subtypes, suggesting NfL has a differential involvement across AD subtypes. However, regional neuro-axonal degeneration patterns in the AD subtypes, and their link to local atrophy patterns are still unclear.

Another feature of interest, which has been shown to correlate with volume loss in AD, is vascular pathology [25, 26]. This involves the degradation of vascular organization and decrease in cerebral blood flow. Previous studies have highlighted microvascular deterioration as a key factor in AD vascular pathology [27, 28]. Here, microvascular health (measured as vessel density, diameter, and wall thickness) was shown to be significantly deteriorated in AD compared to controls, and correlated with cortical volume loss in a region-dependent manner [27, 28]. It is therefore crucial to study the regional differences in microvascular alterations and its association with atrophy between clinical phenotypes, as it could be a valuable marker to distinguish between clinical AD groups.

The current study aims to investigate atrophy patterns in clinically defined AD subtypes and their association not only with regional Aβ and pTau protein accumulation, but also with patterns of neuro-axonal and microvascular degeneration. We hypothesize to find regional differences in atrophy-pathology associations in typical and atypical AD phenotypes.

To investigate these patterns, our study utilized a unique post-mortem cohort of clinically defined and pathologically confirmed AD cases in comparison with non-neurological controls. We determined the atrophy-pathology association through post-mortem in-situ MRI volume measurements and immunohistochemically quantified pathological features of Aβ and pTau protein aggregation, neuro-axonal degeneration, and microvasculature deterioration. Finally, we assessed the translational capability of this study by investigating the agreement between post-mortem and ante-mortem volume measurements.

The results of this study will further characterize the volume-pathology associations and identify possible distinctions between AD clinical phenotypes, which is crucial for future improvements of diagnosis and treatments by attempting to connect atrophy patterns with underlying pathology. This study will further highlight MRI measured volume loss as an important neuroimaging biomarker to assess AD clinical phenotypes in both research and clinical settings.

## Material & methods

### Donor inclusion

In collaboration with the Netherlands Brain Bank (NBB; http://brainbank.nl) we included 33 AD donors, 26 of whom were from the Amsterdam Dementia Cohort [29]. In this cohort, Alzheimer patients were diagnosed as previously described [30]. In short, clinical diagnosis was established through consensus by a multidisciplinary team which included clinical, radiological, genetic and CSF biomarker perspectives. The AD donors can be subdivided into 17 typical and 16 atypical AD donors on the basis of identified initial dominant clinical symptoms, which was reviewed by a clinical neurologist (F.H.B.). The atypical AD cases comprised of three cases diagnosed with the behavioral variant, five with the dysexecutive variant, four with the logopenic variant, and four with the visuospatial variant [5]. Neuropathological diagnosis was confirmed and concomitant pathologies were identified by an expert neuropathologist (A.J.M.R.) and performed according to the international guidelines of the Brain Net Europe II (BNE) consortium [31, 32]. This includes assessment of cytoarchitecture abnormalities and scoring of amyloid, tau, alpha-synuclein and TAR DNA-binding protein 43 (TDP- 43) pathology throughout brain sections sampled from frontal, parietal, temporal, occipital, limbic, brainstem and cerebellar regions. Additionally, 16 age and sex-matched and pathologically confirmed non-neurological controls were selected from the Normal Aging Brain Collection Amsterdam (NABCA; http://nabca.eu) [33]. All donors signed an informed consent for brain donation, and the use of material and clinical information for research purposes. The procedures for brain tissue collection of NBB and NABCA have been approved by the Medical Ethical Committee of Amsterdam UMC (formerly known as VUmc). Available clinical reports of donors were used to obtain clinical dementia rating scores when mentioned.

### Post-mortem in-situ and ante-mortem in vivo MRI acquisition

Post-mortem 3T *in-situ* (brain in cranium) MRI scans were acquired according to a previously described pipeline [33]. Briefly, the following 3T scans (Signa-MR750, General Electric Medical Systems, United States) were acquired with an eight-channel phased-array head-coil: (i) a sagittal 3D T1-weighted fast spoiled gradient echo sequence [repetition time (TR) = 7 ms, echo time (TE) = 3 ms, flip angle = 15°, 1-mm-thick axial slices, in-plane resolution = 1.0 × 1.0 mm^2^, acquisition time = 5 min and seconds]. Moreover, 26 out of 34 AD cases included in our study had ante-mortem in-vivo 3T MRI scans available, see supplementary Table 1 for acquisition details.

### MRI brain volume quantification

Post-mortem and ante-mortem 3D T1 images were automatically segmented using QyScore® software, developed by Qynapse (https://qynapse.com/qyscore, [34, 35]). The QyScore® platform utilizes a combination of algorithms including voxel wise probabilistic modeling for tissue type classification (gray matter, white matter, and CSF), a competitive region growing algorithm (hippocampus and amygdala for this study), and convolutional neural network deep learning approaches to provide quantitative measures for additional subcortical regions, brainstem, and cerebellum [36].

T1-weighted 3D MR images underwent N4 bias-correction for contrast inhomogeneity, tissue type classification was modelled on these corrected tissue intensity profiles and neuroanatomical probability maps. Spatial normalization was performed in two stages to account for morphological variability: first, an affine transformation aligned images to MNI152 space, followed by a nonlinear registration for fine-scale anatomical alignment. GM probability maps were thresholded based on individual corrected tissue intensity profiles to create binary masks, which were then non-linearly spatially matched to the AAL3 atlas [37] in MNI152 space using nearest-neighbor mapping for discrete cortical region labeling.

The quantitative measurements of the global, lobular and subcortical atrophy were expressed either as raw volumes (ml), as a percentage of the intracranial volume (%ICV), or as population-normed z-scores and percentiles resulting from the comparison to a large database of healthy age- and sex-matched controls. The AAL3 brain atlas regions were provided in this study as raw volumes (ml). Correctness of anatomical boundaries of segmentations and parcellations masks were manually quality checked for each MR image. Additionally, visual MRI scores (Global cortical atrophy, parietal cortical atrophy, medial temporal lobe atrophy and Fazekas score) were determined by a clinical radiologist (F.B.).

### Tissue Sampling

After MRI acquisition, autopsy was performed, all within 12 h after death. Fixated right brain hemispheres (four weeks in 4% buffered PFA) were cut into 1 cm sections and dissected into tissue blocks by a neuropathologist (A.J.M.R.) for the NBB AD cases and by a neuroanatomist (W.v.d.B.) for the NABCA control cases. Tissue was subsequently paraffin-embedded as previously described [33]. The following regions of the right hemisphere were included for the current study: middle frontal gyrus (GFM), middle temporal gyrus (GTM), superior parietal gyrus (GPS), posterior cingulate gyrus (PCC), precuneus (Precun), primary visual cortex of the occipital cortex (OC), hippocampus (Hip; tissue block also includes the entorhinal and parahippocampal gyrus).

### Immunohistochemistry

Six µm sections from the above-mentioned regions were cut and mounted on superfrost + glass slides (Thermo Scientific USA). Sections were stained for Aβ (clone 4G8, Biolegend USA, 1:8000), pTau (clone AT8, Thermo Scientific USA, 1:800), NfL (NfL, Synaptic Systems 1:600) and Collagen IV (COLIV, Abcam UK, 1:2000). Sections underwent heat induced epitope retrieval (HIER) for 30 min in a steamer in pre-heated 10 mM Citrate Buffer pH 6.0 (4G8, AT8 and COLIV) or 10 mM Tris–EDTA buffer pH 9.0 (NfL). The sections were blocked for endogenous peroxidase using 0.3% hydrogen peroxide in tris buffered saline (TBS; pH 7.6) and consequently blocked for non-specific binding using 1% normal goat serum and 0.5% Triton X. Primary antibodies were diluted in blocking serum and incubated overnight at 4 °C. Primary antibodies were detected using EnVision (Dako, Glostrup, Denmark), and visualized using 3.3’-Diaminobenzidine (DAB, Dako) with Imidazole (50 mg DAB, 350 mg Imidazole and 1 μl of H2O2 per 100 ml of Tris–HCl 30 mM, pH 7.6). Between steps, TBS was used to wash the sections. After counterstaining with haematoxylin, the sections were dehydrated and mounted with Entellan (Merck, Darmstadt, Germany).

### Pathological quantification

Images were taken using a whole-slide scanner (Vectra Polaris, 20 × objective) and quantified using QuPath Version 4.3 (https://qupath.github.io/). Regions of interest containing all cortical layers were delineated in straight areas of the cortex, to avoid over- or underestimation of pathology in sulci and gyri, respectively [38]. Hippocampus sections were segmented into hippocampus proper (including dentate gyrus, cornu ammonis (CA) 1–4, subiculum and parasubiculum) and parahippocampal regions (entorhinal cortex and parahippocampal gyrus combined) according to previously anatomical characteristics, and to match MRI annotated regions [39, 40].

Using a QuPath pixel classifier function, a model was trained on a representative subset of the data to quantify the DAB positive signal. For each immunohistochemical (IHC) marker, the outcome measure was the % of immunoreactivity per area of interest (area%) for each brain region. For microvasculature wall thickness and vessel area quantification, horizontally cut vessels were automatically isolated according to vessel shape characteristics, and manually quality checked with a minimum of at least three vessel detections per section. Wall thickness was quantified using FIJI 1.54 [41] with the vessel analysis plugin (version 1.1) adapted for an automated pipeline for the current data. Vessel area was estimated using area measurements of the best rectangular fit to the vessel. Vessel wall thickness and the square root of the vessel wall area were then used to create the wall thickness/area ratio measurement.

**Fig. 1:**
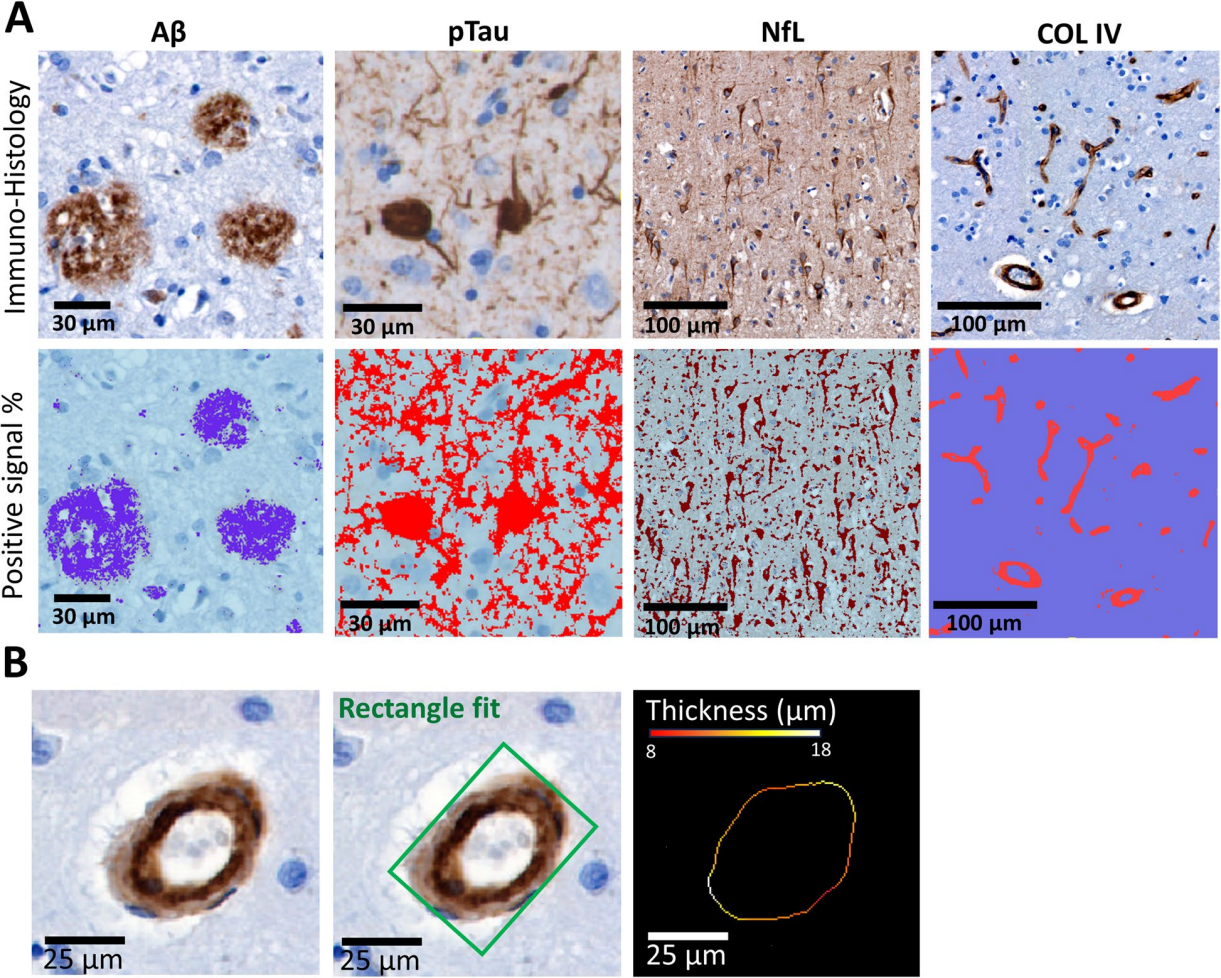
Quantification of pathological markers. **A** Examples of imaged pathology and matching quantification results for Amyloid-β, pTau, NfL and COLIV. Positive signal % over a given area is quantified as area %. **B** Examples of best rectangle fit and vessel wall thickness measurements in COLIV positive vessels

In summary, the IHC outcome measures were immunoreactivity (area%) of Aβ, pTau, NfL and COLIV, as quantification of pathological load, neuro-axonal degeneration, and microvascular density (Fig. 1). For microvascular density, additional outcome measures were vessel wall thickness, vessel area, and the thickness/area ratio.

### Semi-quantitative scoring of Aβ plaques

To assess the heterogeneity in Aβ plaque deposits, such as diffuse, classic, coarse grained, subpial, perivascular and cerebral amyloid angiopathy (CAA, type 1 or type 2) Aβ depositions [42, 43], a semi-quantitative analysis of Aβ plaque morphology was performed. Each section was assessed at a (digital) magnification of 100x. coarse-Aβ plaque morphologies were scored on a “CERAD-like” 4-point scale: 0 = none; 1 = very sparse (limited to moderate amount [2–5] of localized depositions, or a few sporadic singular depositions throughout the cortical region); 2 = sparse (moderate amount of depositions in several areas throughout the cortical region); 3 = frequent (depositions covering (almost) the entirety of cortical area, subpial area or vessels > 75%) [44]. Scoring was performed blinded to case identity over the entire section (N.R.). A second assessor (L.J.) performed scoring on a subset of cases to assess intra-rater and inter-rater agreement.

### Statistical analysis

Statistical analyses were performed in Rstudio Version 4.3.2. Group differences between typical AD, atypical AD and controls were assessed. Subsequent analysis of clinical subtypes within the atypical phenotype group could only be approached using exploratory analysis without statistical testing due to small sample sizes. Cohort characteristics were analyzed using linear regression models for continuous data, and Fisher’s exact test for categorical data. All AAL3 atlas regional volume differences between typical AD, atypical AD and controls were assessed using linear regression models and presented as Cohen’s D effect sizes. Due to large volume differences between regions, brain volumes were scaled to the mean of the control group for each region, which was set at 100%. For both MRI volume (lobar and selected regions) and immunohistological measurements, group differences between typical AD, atypical AD and controls were assessed using linear mixed models with a random intercept for subjects, with an added regional interaction term when analyzing regional differences. Post-hoc analysis were performed using the models estimated marginal means. Semi-quantitative scores of amyloid morphology frequency were assessed using Fisher’s exact test. The strength of the inter-rater and intra-rater agreement was tested using the intraclass correlation coefficient (ICC), and magnitude of effect size was interpreted according to suggestions described by Koo & Li [45],ICC < 0.5 = poor, 0.5 < ICC < 0.75 = moderate, 0.75 < ICC < 0.9 = good, ICC > 0.9 = excellent. Volume-pathology associations were assessed using linear mixed models with a groupwise interaction term and random intercepts for subjects, a regional interaction term was added when analyzing regional associations. Standardized regression coefficients (βs) were calculated with a model refit which used the standardized version of the data, processed using the “parameters” R package, improving interpretability by allowing a one-unit increase in a variable to be equal to its standard deviation. A p-value of ≤ 0.05 was considered significant throughout all analyses and were corrected for multiple comparison testing using the false discovery rate (FDR) method in a groupwise fashion (e.g. control, typical and atypical AD). All analyses including pathological data were corrected for age at death and sex; all analyses including MRI data were corrected for age at death, sex, post-mortem delay (PMD) and intracranial volume (ICV).

## Results

### Cohort characteristics

Demographical, clinical, pathological, and radiological characteristics of included donors are summarized in Table 1 and in detail per case in supplementary Table 2. An apparent lower percentage of females was observed in both typical AD (− 50%) and atypical AD (− 37.5%) compared to controls, but this difference in sex proportion was not significant. Both age at death and post-mortem delay did not differ between control and both AD groups. Clinical dementia rating (CDR) scores did not differ between typical and atypical AD. Atypical AD cases showed a shorter disease duration compared to typical AD (*p* = 0.001). The atypical AD group showed a higher occurrence of APOE4 carriers compared to controls (*p* = 0.032). By definition, AD pathological scores of Braak NFT stage, Thal Aβ phase and CAA-type were higher in both AD groups when compared to controls (all *p* < 0.001). Braak Lewy Body (LB) stage did not differ between both AD and control groups. Furthermore, normalized whole brain volume (typical AD: − 13.25%, *p* < 0.001; atypical AD: − 12.96%, *p* < 0.001), gray matter volume (typical AD: − 10.7%, *p* < 0.001; atypical AD: − 13.18%, *p* = 0.002), and white matter volumes (typical AD: − 16.67%, *p* < 0.001; atypical AD: − 12.67%, *p* = 0.002) were lower in both AD groups compared to controls. Medial temporal lobe atrophy score was higher in the AD groups compared to controls (typical AD: *p* = 0.002; atypical AD: *p* = 0.002), and the Fazekas score was higher in the typical AD group (*p* = 0.016) but not in the atypical AD group when compared to controls.

**Table 1:** Cohort characteristics

	**Control**	**Typical AD**	**Atypical AD**
	*n* = 16	*n* = 17	*n* = 16
**Sex** (Female)	8 (50%)	4 (23.5%)	5 (31.2%)
**Age at death** (In years)	71 (± 8) [59–85]	69 (± 11) [53–89]	66 (± 10) [37–78]
**Age at Onset** (In years)	NA	61 (± 10) [46–81]	61 (± 10) [32–74]
**Disease duration** (In years)	NA	8 (± 3) [2–14]	4 (± 3)^ [1–10]
**Clinical Dementia Rating Scale** NA/0/1/2/3	NA	6/0/5/2/4	2/0/3/2/9
**Clinical Subtyping** NA/Behav/dysex/logo/visuo	NA	NA	1/3/5/3/4
**APOE genotyping** APOE4 carrier	4 (26.6%)	10 (58.8%)	11 (68.8%)*
**Post-Mortem delay** (Hours:minutes)	8:13 (± 1:51)	8:12 (± 1:25)	7:42 (± 1:46)
**MRI**			
**Normalized Whole Brain Volume** % ICV	70.2 (± 4.5)	60.9 (± 5.6)***	61.1 (± 7.7)***
**Normalized Gray Matter Volume** % ICV	40.2 (± 3.3)	35.9 (± 3.3)***	34.9 (± 5.5)**
**Normalized White Matter Volume** % ICV	30.0 (± 1.9)	25.0 (± 4.3)***	26.2 (± 4.1)**
**Global Cortical Atrophy** 0/1/2/3/4	13/3/0/0	2/10/4/1***	5/6/2/3*
**Parietal Cortical Atrophy** 0/1/2/3/4	10/5/1/0	0/9/6/2***	1/4/8/3***
**Medial Temporal Lobe Atrophy** 0/1/2/3/4	9/6/1/0/0	1/5/4/4/3**	1/5/4/5/1**
**Fazekas** 0/1/2/3	6/3/7/0	6/5/1/5*	4/3/6/3
**Pathology**			
**Braak NFT stage** 0/1/2/3/4/5/6	3/10/3/0/0/0/0/	0/0/0/0/3/5/9 ***	0/0/0/0/1/6/9 ***
**Thal Aβ phase** 0/1/2/3/4/5	3/7/4/2/0/0	0/0/0/1/0/16 ***	0/0/0/0/2/14 ***
**Braak LB stage** 0/1/2/3/4/5/	14/2/0/0/0/0/	14/0/1/0/1/1/	13/0/0/1/0/2
**LATE stage** 0/1/2/3	16/0/0/0	11/2/3/1	13/1/1/1
**CAA-Type** 0/1/2	13/1/2	1/14/2 ***	0/10/6 ***

Data is noted as mean (± standard deviation) [range] or count (ratio %)

*NA* not available or not applicable, *behav* behavioral subtype, *dysex *dysexecutive subtype, *logo* logopenic subtype, *visuo* visuospatial subtype *NFT *Neurofibrillary Tangle, *LB *Lewy Body, *LATE *Limbic-predominant Age-related TDP- 43 Encephalopathy, *ICV* Intracranial Volume

Significance between control and AD phenotypes is denoted with * = *p* ≤ 0.05, ** = *p* ≤ 0.01, *** = *p* ≤ 0.001 and between AD groups similarly with^

### MRI measured brain volume

When assessing lobar volume, compared to controls, both AD phenotype groups showed lower volume in the temporal (typical AD: -11.42%, *p* = 0.007; atypical AD: %, *p* = 0.007), insular (typical AD: -14.31%, *p* = 0.030; atypical AD: -22.91%, *p* = 0.002) and limbic lobes (typical AD: -7.57%, *p* = 0.005; atypical AD: − 8.10%, *p* = 0.005), only the typical AD group showed a lower parietal lobe volume (-11.11%, *p* = 0.042) (supplementary Table 3). There was no observed difference in lobar volume between typical and atypical AD. Variation between atypical subtypes was observed to be most pronounced in the insular lobe (supplementary Fig. 1).

**Fig. 2:**
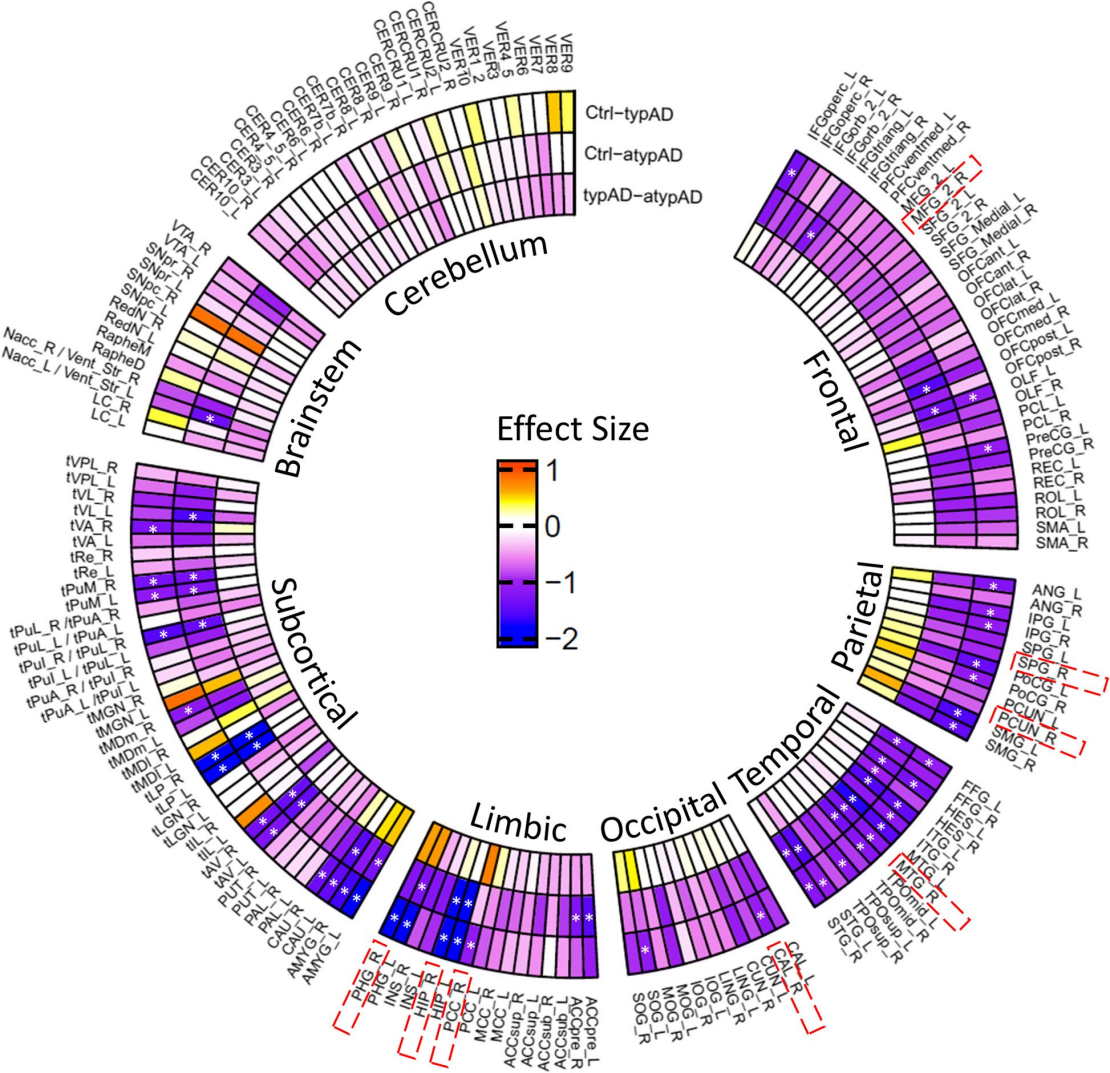
Group Comparisons of regional volume between controls and clinical AD phenotype groups. AAL3 atlas regions are grouped by lobes and brain structures and sorted in alphabetical order. Comparisons in order from outer ring to inner ring: control vs typical AD, control vs atypical AD, typical AD vs atypical AD. Volume differences are displayed as Cohen's D effect sizes, with negative effect size (blue) denoting a lower volume in the second group of each paired comparison. Significant group differences are annotated with * = *p* ≤ 0.05, corrected for multiple comparisons. Highlighted in red boxes are regions selected for association with pathological quantification

Differences between typical AD, atypical AD, and controls were explored for all AAL3 atlas regions are shown in Fig. 2. Both AD groups showed a generally lower volume compared to controls, most pronounced in limbic, parietal, and temporal regions. AD phenotype groups showed only minor differences, primarily in limbic regions, which were not significant. See supplementary Table 4 for all regional significant differences after multiple comparison.

**Fig. 3:**
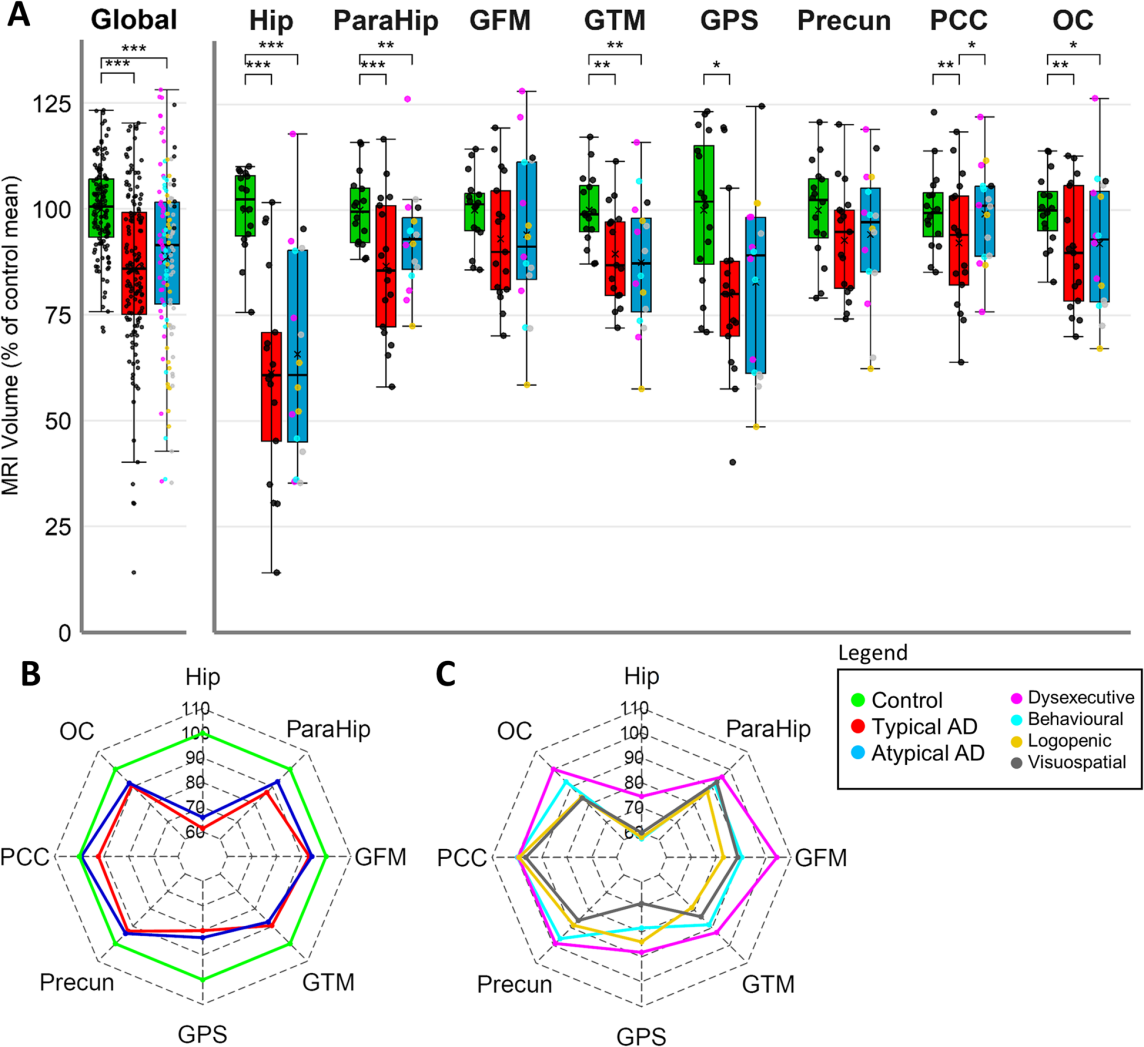
Global and regional cortical MRI volume of selected regions. Global data indicates all eight selected regions combined. **A** boxplots of cortical MRI volume as % of control mean, which was set at 100% for each region. **B** Radar plot of clinical phenotypes visualizing control, typical and atypical AD. **C** Radar plot visualizing atypical subtypes, namely dysexecutive, behavioral, logopenic and visuospatial groups. Radar plots denote the mean volume for each region per group. * = *p* ≤ 0.05, ** = *p* ≤ 0.01, *** = *p* ≤ 0.001. Hip = hippocampus, ParaHip = parahippocampal gyrus, GFM = middle frontal gyrus, GTM = middle temporal gyrus, GPS = superior parietal gyrus, Precun = precuneus, PCC = posterior cingulate cortex, OC = occipital cortex

In addition to the analysis of all AAL3 atlas regions analysis, we assessed volume differences restricting to brain regions used for histopathological quantification (Fig. 2 regions with red box), where both AD groups showed lower overall cortical volume when compared to controls (typical AD: -14.17%; atypical AD: -11.4%, both *p* < 0.001), see Fig. 3A. Regionally, the hippocampus (typical AD: *-*38.63%; atypical AD: -34.15%, both *p* < 0.001), parahippocampus (typical AD: − 13.3%, *p* < 0.001; atypical AD: − 7.11%, *p* = 0.003), middle temporal gyrus (typical AD: − 10.39%; atypical AD: − 12.45%, both *p* = 0.003), and occipital cortex (typical AD: − 9.35%, *p* = 0.005; atypical AD: -7.95%, *p* = 0.017) showed lower volume in both AD phenotypes compared to controls. The superior parietal gyrus showed a lower volume in typical AD compared to controls (− 19.92%, *p* = 0.018), which was not observed in the atypical group. The posterior cingulate gyrus had a lower volume in typical AD compared to atypical AD (-6.93%, *p* = 0.043) and controls (-7.76%, *p* = 0.005). Exploring the subtypes within the atypical AD group, the behavioral, logopenic and visuospatial group subtypes showed an overall lower regional volume compared to the dysexecutive group, especially in the hippocampus (-20.53% on average), middle frontal gyrus (-14.95% on average) and occipital cortex (-12.24% on average) (Fig. 3C and supplementary Table 3). In addition, the visuospatial group showed an overall lower regional volume compared to the average of the other AD subtypes, especially evident in the superior parietal gyrus (-17.09% on average). Correlations between MRI measured volume and visual assessed atrophy scores showed good concordance, see supplementary Table 5.

### Histopathological load and distribution

**Fig. 4:**
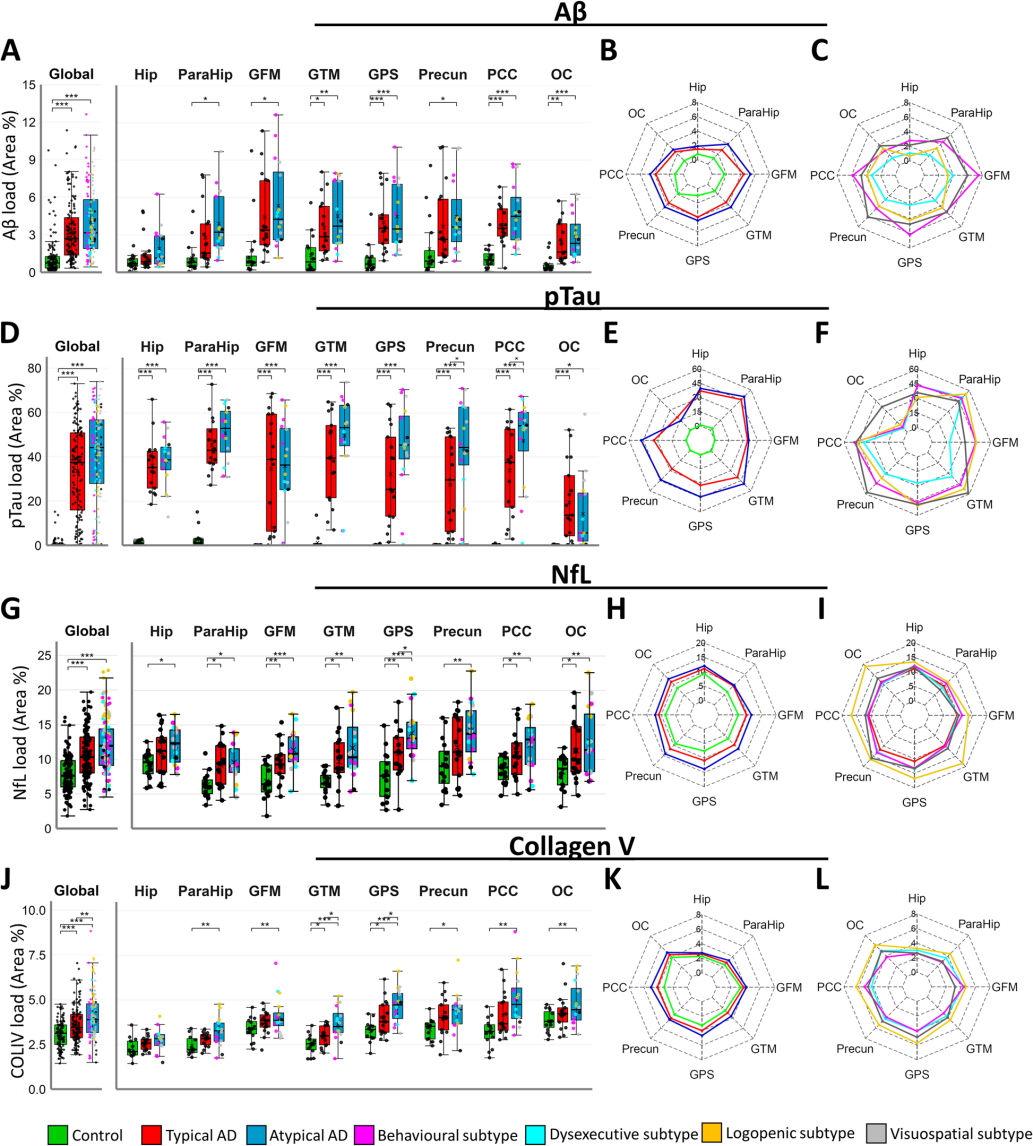
Pathological load of cortical Aβ, pTau, axonal damage and microvascular deterioration in AD phenotypes and controls. Boxplots of immunoreactivity of histological markers Amyloid beta (**A**) pTau (D) NfL (**G**) and COLIV (**J**) as area% load in selected brain regions in control and AD phenotype groups (behavioral, dysexecutive, logopenic and visuospatial). Radar plots showing the distribution of pathology load among control, typical AD, atypical AD (**B**, **E**, **H**, **K**), and distinct subtype groups (**C**, **F**, **I**, **L**). * = *p* ≤ 0.05, ** = *p* ≤ 0.01, *** = *p* ≤ 0.001. Hip = hippocampus, ParaHip = parahippocampal gyrus, GFM = middle frontal gyrus, GTM = middle temporal gyrus, GPS = superior parietal gyrus, Precun = precuneus, PCC = posterior cingulate cortex, OC = occipital cortex

Global load of all pathological markers (Aβ, pTau NfL and COLIV) was higher in both AD phenotype groups compared to controls (all *p* < 0.05, Fig. 4A, D, G, J). There was a pattern of higher global pathological load in atypical AD compared to typical AD for all markers, but this was only significant for COLIV (+ 13.66%, *p* = 0.040) (Fig. 4J).

Regionally, the load of pathological markers was higher in atypical AD compared to typical AD in several areas. pTau load was increased in the precuneus (+59.91%, *p* = 0.018) and posterior cingulate cortex (+39.44%, *p* = 0.048). NfL levels were elevated in the middle temporal gyrus (+20.08%, *p* = 0.015), while COLIV load was higher in the superior parietal gyrus (+27.00%, *p* = 0.037 and +17.09%, *p* = 0.033). Differences in load and distribution between atypical subtypes were examined in an exploratory observational approach, revealing a distinctly higher pTau load in the occipital cortex in the visuospatial phenotype compared to all other subtypes (Fig. 4F, with additional visual details in Supplementary Fig. 2). A comprehensive overview of mean pathological marker load for each group is available in Supplementary Table 3.

No significant regional differences for Aβ were observed between AD phenotypes. Semi-quantitative analysis of Aβ plaque morphology revealed a higher global classical cored plaque frequency in the atypical AD group compared to typical AD across all regions (*p* < 0.001). However, regionally no significant differences between typical and atypical AD groups were found (supplementary Fig. 3).

Further investigation of microvascular deterioration was done by analyzing the ratio value between the vessel lumen (vessel area) and the vessel wall diameter. No difference between groups were observed (supplementary Fig. 4 A), except for the precuneus where both AD phenotype groups showed a lower mean ratio value than controls (typical AD: -14.27%; atypical AD -15.18%, both *p* = 0.003). Exploratory analysis indicated regional variability in ratio between atypical subtypes (supplementary Fig. 4B). A significant negative correlation was found between ratio value and Aβ load across whole AD group (r = -0.145*, p* = 0.050), which was driven by atypical AD (r = -0.290*, p* = 0.005) (supplementary Table 6). Additionally, ratio value showed a negative association with CAA severity score in both typical (r = -0.536*, p* < 0.001) and atypical AD (r = -0.353*, p* < 0.001) (supplementary Table 7).

### Association between MRI volume and pathological markers

**Fig. 5:**
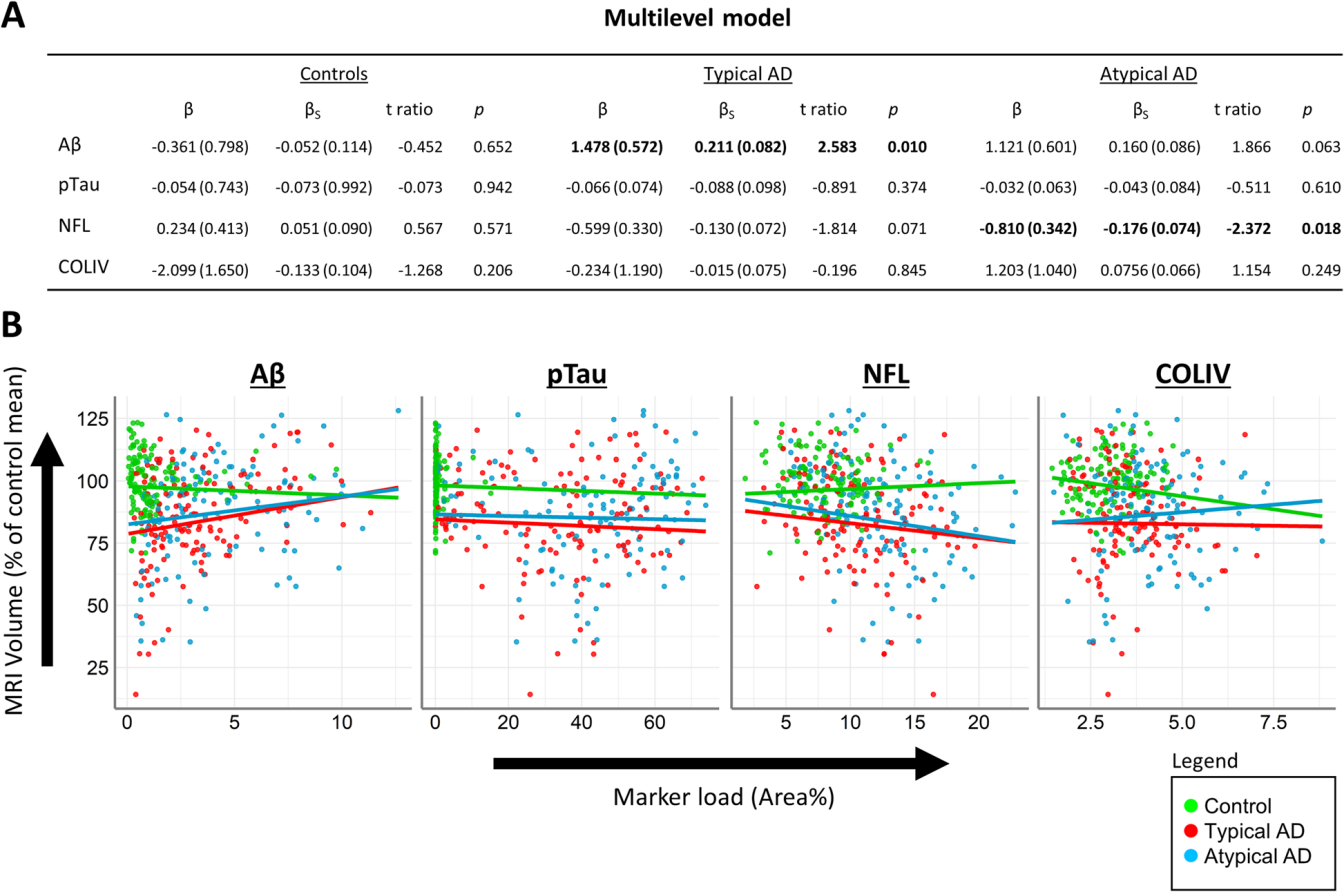
Global volume-pathology associat*ions for AD clinical phenotypes.* **A** Results of multilevel model for control and AD phenotype groups for each immunohistological marker. Data are presented as β (± SE) for volume change rates or as β_s_ (± SE) for standardized volume change rates. Significant results are displayed in bold. **B** Scatter plots and regression slopes of each immunohistological marker for controls and AD phenotype groups

When assessing global associations (i.e., across all selected regions) a positive association between volume and Aβ load was found in both AD phenotypes, but the effect was only significant in the typical AD group (typical: βs = 0.211, *p* = 0.010; atypical: βs = 0.160, *p* = 0.063). A negative association between global volume and NfL load was found in both AD phenotypes, but the effect was only significant in the atypical AD group (typical: βs = − 0.130, *p* = 0.071; atypical: βs = − 0.176, *p* = 0.018). No significant associations were found between global volume and pTau or COLIV load, nor for the control group with any marker (all p > 0.05, see Fig. 5 for all results).

**Fig. 6:**
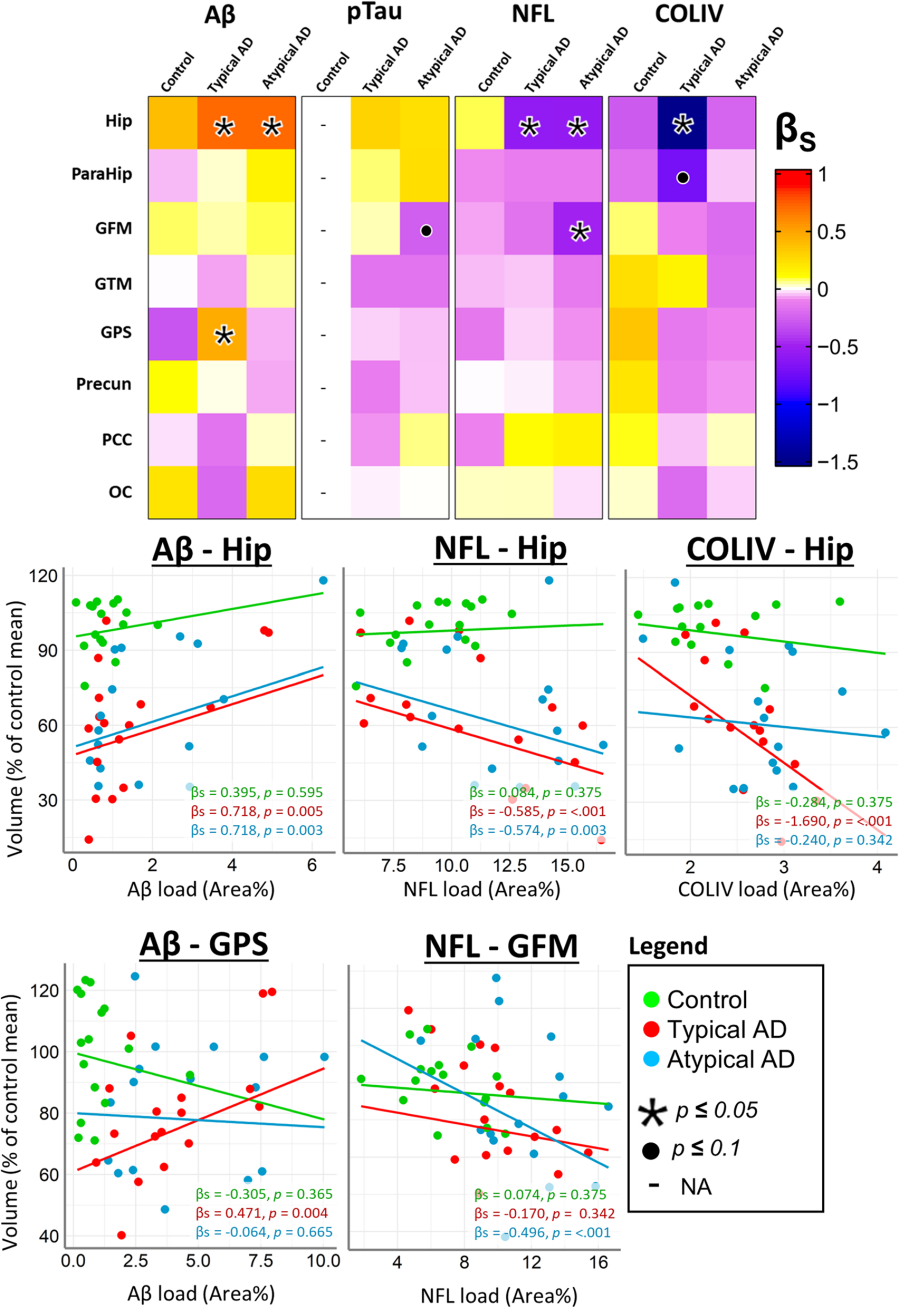
Regional Volume-Pathology associations for AD clinical phenotypes. Standardized betas of volume-pathology associations are presented in a heatmap to provide a better overview of all regional associations. For each significant association, scatterplots with regression lines are plotted. By definition, control cases have little-to-no pTau load in the selected regions therefore control volume-pathology associations for pTau are not included and are designated by the – sign. Hip = hippocampus, ParaHip = parahippocampal gyrus, GFM = middle frontal gyrus, GTM = middle temporal gyrus, GPS = superior parietal gyrus, Precun = precuneus, PCC = posterior cingulate cortex, OC = occipital cortex

A detailed overview of regional associations between volume and pathology can be found in Fig. 6 and supplementary Table 8. In summary, a positive association between volume and Aβ load was found in the hippocampus for both clinical AD phenotypes (typical: βs = 0.718, *p* = 0.005; atypical: βs = 0.718, *p* = 0.004), and in the superior parietal gyrus in typical AD (βs = 0.471, *p* = 0.004). No significant associations were found between regional volume and pTau load after correction for multiple comparisons. Hippocampal volume associated negatively with NfL load in both clinical AD phenotypes (typical: βs = -0.585, *p* < 0.001; atypical: βs = -0.574, *p* = 0.004). In the middle frontal gyrus, a negative association with NfL load was found in atypical AD (βs = -0.496, *p* = 0.011). Exploring this data further, no specific atypical subtype showed to be driving this effect (supplementary Fig. 5). Hippocampal volume negatively associated with COLIV load, only in typical AD (βs = -1.691, *p* < 0.001). To further assess this association, the hippocampi of typical AD cases were visually examined for COLIV immunoreactivity, revealing an observed increase in COLIV-positive vessel count (supplementary Fig. 6).

### Post-mortem MRI as proxy for ante-mortem MRI

**Fig. 7:**
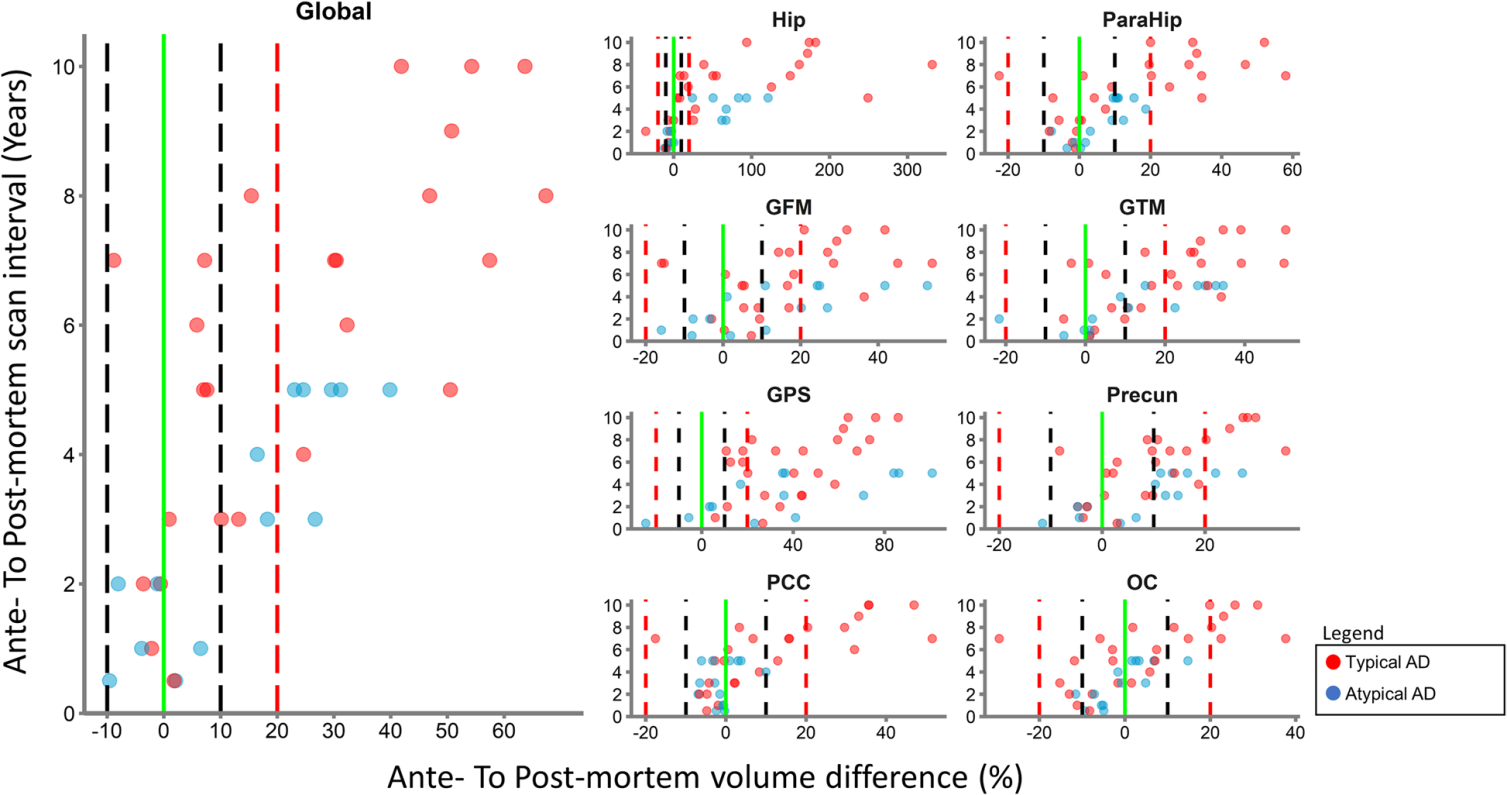
Scatter plot ante-mortem to post-mortem scan interval and volume difference. Each data point represents a comparison between the ante- and post-mortem scan of a given case. Vertical lines are drawn at 0% (green) − 10% and 10% (black) and at − 20% and 20% (red) volume difference to indicate no, small, and large volume differences, respectively. Time difference between ante and post-mortem interval is presented as ante- to post-mortem interval. Volume difference was calculated as the percentage difference between ante-mortem to post-mortem scan volume. Hip = hippocampus, ParaHip = parahippocampal gyrus, GFM = middle frontal gyrus, GTM = middle temporal gyrus, GPS = superior parietal gyrus, Precun = precuneus, PCC = posterior cingulate cortex, OC = occipital cortex

To assess the coherence of post-mortem *in-situ* MRI with ante-mortem in vivo MRI derived brain volumes, global and regional differences of ante to post-mortem scan volume was assessed. At a scan interval within two years, 100% of cases had an ante- to post-mortem global (i.e., volume across all studied regions) volume difference below 10%. At intervals higher than two years, the majority (62% of cases) had a global volume difference exceeding 20% (Fig. 7). When assessing the regional volume of ante- to post-mortem differences, most regions showed small differences (< 10%) at a scan interval within two years, including the hippocampus. However, the superior parietal gyrus showed a larger (< 20%) difference within two years in 40% of cases. This percentage increases to 80% of cases at scan intervals above two years. The hippocampus showed the largest volume differences at longer time intervals, ranging up to an ante- to post-mortem difference of 320% (Fig. 7).

## Discussion

In this post-mortem MRI and immunohistochemistry study, we investigated the association between MRI atrophy patterns and immunohistochemical markers for Aβ and pTau aggregation, neuro-axonal damage, and microvascular alterations, in patients with clinical AD phenotypes. Using MRI-measured volume, we observed a global positive association with Aβ and a negative association with NfL, with minimal differences across AD clinical groups. Furthermore, regional differences in volume-pathology associations between phenotypes were observed: only in typical AD, a higher superior parietal gyrus volume was associated with higher Aβ load, and a lower hippocampal volume with higher COLIV load; only in atypical AD, a lower middle frontal gyrus volume was associated with higher NfL load.

As expected, compared with controls, AD donors presented decreased cortical volume, specifically in the limbic, temporal, parietal and occipital regions. While we did find parietal atrophy selectively in typical AD, clinical AD phenotype groups did not show distinctly different patterns of atrophy as would be expected on the basis of the literature (e.g. less hippocampal or more pronounced frontal atrophy in atypical AD) [4, 5, 46, 47]. An explanation could be the advanced AD disease stage of our cohort; in early stages, atrophy and pathology patterns might be selective to specific brain regions [46, 47], whereas more diffuse brain atrophy occurs at later stages of the disease [1, 2, 48], masking the initial regional volume differences between phenotypes.

However, a lower posterior cingulate cortex volume was observed in typical AD compared to atypical AD. This highly connected region plays a key role in autobiographical memory and multiple brain networks and is often affected early in AD, particularly in relation to default mode network disruptions and changes in metabolic activity [49–51]. Notably, dysconnectivity in AD is increasingly recognized as a key driver of disease progression, possibly even shaping distinct AD phenotypes, with the posterior cingulate potentially playing a crucial role in these network disruptions [52, 53]. Furthermore, previous findings indicate that posterior cingulate pathology as well as atrophy, can differentiate between typical AD and other dementias, such as patients within the spectrum of frontotemporal dementia (FTD), particularly semantic dementia [54, 55]. Here, evidence of posterior cingulate volume differentiating between phenotypes was found. Whether the lower posterior cingulate volume is due to the progression of the parietal atrophy pattern in typical AD, network-mediated degradation effects, or other underlying mechanisms, requires further investigation.

Compared to typical AD, the atypical AD cases in our cohort not only had a shorter disease duration, as was expected [5], but also a greater load of all pathological markers included in our study. These findings suggest a greater accumulation of pathological hallmarks, neuro-axonal and microvascular degeneration within a shorter time frame, indicative of a more aggressive disease manifestation in atypical AD.

In terms of cortical volume and pathology associations, the positive association between Aβ and volume found in the current study has been previously described, but instead in early stages of AD [56–60]. Similar associations, but instead with cortical thickness, were found previously by our group in a subset of cases [61]. Here it was hypothesized that the space encompassing process of plaque formation was captured by the high resolution quantification of Aβ because of the immunohistochemical approach. More recently, anti-amyloid therapies showed a decrease in (PET and CSF measured) Aβ levels associated with a loss of brain volume, spawning the hypothesis that Aβ mediated inflammation induces swelling of brain tissue, subsequent clearance of Aβ reduces this swelling [62–64]. Although the mechanism is still not clear, the results in this study provide evidence that Aβ accumulation could be contributing, whether directly or indirectly, to a volume increase in the cortex. Aβ-mediated volume increase appears strongest in the hippocampus and superior parietal gyrus in typical AD cases. These regions also exhibit the largest volume differences compared to controls, suggesting a potentially counterintuitive relationship in which Aβ-related volume increase is most pronounced in areas most vulnerable to neurodegeneration. However, further research is needed to uncover the underlying mechanisms.

The absence of an association between volume and pTau pathology in this study is in contrast with previous findings in which Tau PET tracer uptake strongly aligns with brain atrophy and clinical presentation [12, 13, 65, 66]. In this study tangles and neuropil threads were not separately quantified, which might have affected the ability to detect volume associations, as tangles are more indicative of neurodegeneration [67–69]. Additionally, the antibody used to detect pTau in this study (AT8) is effective at identifying tangle isoforms prominent in early to intermediate stages of pathological maturation, but less effective at detecting the most advanced isoform, the so-called ghost tangles [68, 70]. A better detection of these advanced isoforms combined with improved isolation of tangle morphologies could better reflect previous findings.

In the current study, neuro-axonal damage (increasing NfL due to degradation of neurons and axons [71]) showed to be a strong indicator for overall brain volume loss for both clinical AD phenotypes, most pronounced in the hippocampus. This replicates effects previously found in CSF and plasma studies, in which NfL levels strongly associated with brain atrophy [19–23]. A higher NfL load was associated with lower middle frontal gyrus volume only in atypical AD, possibly indicating specific regional vulnerability to axonal damage in the atypical phenotype. However, this requires further validation in a larger independent atypical subtype(s) cohort.

The strongest regional association observed in this study was between decreased hippocampal volume and higher COLIV load. This increase in hippocampal COLIV load was not due to thickening of the vascular wall as expected, but due to an apparent increase in vascular density. This effect could be due to a significant loss of brain volume combined with a maintenance, or at least a relatively low decrease, of vascular density, resulting in a net increase of blood vessels. Previous research also points towards angiogenesis and subsequent hypervascularization as response to impaired tissue perfusion and inflammation in AD [72–74]. Nevertheless, this response was only observed in the hippocampus of typical AD cases, hinting at a unique response of vascular density to volume loss in typical AD.

To build on previous studies observing good comparability between post-mortem and ante-mortem MRI for cortical thickness [61, 75], this study also showed good comparability for cortical volume. At short ante-to-post-mortem scan intervals, i.e. within 2 years, the volume differences were less than 10%. This finding validates structural post-mortem MRI as a proxy for structural ante-mortem MRI measurements and facilitates the possible translation of pathological findings towards a clinical setting via MRI outcome measures. Hippocampal ante-to-post-mortem volume differences were exceptionally large at higher scan intervals, showing that disease progression strongly affects hippocampal volume. Relative to other cortical regions, the superior parietal gyrus showed the greatest ante-to-post-mortem volume differences, indicating this region might be more affected by disease progression than other cortical regions. Studies combining ante-mortem MRI with neuropathology should be aware of these regional differences as they can affect interpretation of their results.

The strengths of this study include the unique cohort and pipeline setup, including clinical phenotype information, post-mortem and ante-mortem MRI data, and immunohistological quantification of pathological hallmarks and other markers of neurodegeneration in multiple brain regions. However, there were also some limitations. While clinical dementia rating scores still indicate a variability in clinical disease stage, neuropathologically all cases showed to be highly burdened (Braak NFT stage V and VI), which may have influenced the lack of different typical and atypical atrophy patterns often reported in the literature. Additionally, no significant sex and age differences were observed between groups, but the effect may not have been detectable due to the limited sample size. Another limitation is the grouping of atypical subtypes into one atypical phenotype group due to small sample sizes of subgroups, possibly introducing variability and masking subtype specific effects. Furthermore, recent studies highlight the difficulty of diagnosing clinical phenotypes and show not only symptomatic variability within a given phenotype but also overlap in symptoms between phenotypes [76, 77]. In turn, atrophy and pathology patterns can be expected to show heterogeneity within and similarities between phenotypes, making it difficult to detect differences between groups. Although we included markers of neurodegeneration such as neuro-axonal and microvascular degeneration, there are several other markers of interest which may influence regional cortical volume. For instance, neuroinflammatory response has recently been found to differ between clinical phenotypes in a regional manner, which could also be linked to atrophy [78]. In addition, loss of synapses or of specific neuronal and glial cell types have a strong effect on regional volume, which might differ between clinical AD subtypes [79–81]. Similarly, more advanced imaging methods that are more sensitive to a variety of neuropathological changes and group differences could be applied, such as the T1-weighted/T2-weighted ratio which could be applicable for capturing myelin degradation in the cortex [82, 83], or diffusion-weighted imaging approaches which can assess deterioration of fiber tracts or cortical tissue integrity [84]. Investigating other theories explaining clinical and pathological heterogeneity may also be valuable. For instance, the probabilistic model of AD which puts decreasing weight on the amyloid pathophysiological cascade and increasing weight on environmental factors and risk genes as biological underpinnings, could be worth pursuing in future studies [85].

## Conclusion

This study shows that cortical volume in AD is predominantly influenced by Aβ and neuro-axonal degeneration, albeit in opposing directions, and that region-specific distinctions in atrophy-pathology associations can be observed between typical and atypical AD. While using cortical volume as an imaging marker to distinguish between AD phenotypes remains challenging, understanding its relationship with the underlying pathological features is crucial for enhancing the interpretation of AD MRI data sets in heterogeneous cohorts in both research and clinical settings.

## Supplementary Information

Supplementary Material 1. Supplementary Table 1. Ante-mortem MRI scan specifications. Supplementary Table 2. Cohort characteristics per case. Supplementary Table 3. Cortical Volume and Pathological load per Phenotype group and subtypes. Supplementary table 4. Significant results from group comparisons in regional volume of AAL3 regions. Supplementary Table 5. Correlation analysis between MRI volume measurements and visual atrophy scores. Supplementary Table 6. Correlation analysis between pathologies. Supplementary Table 7. Correlation analysis between CAA and Aβ load, COLIV load and ratio. Supplementary Table 8. Linear mixed model results regional volume pathology associations.

Supplementary Material 2. Supplementary Figure 1. MRI lobular volume results. A) boxplots of lobular volumes across all cortical lobes of each group. Volumes are presented in boxplots as % of control mean volume which was set at 100% for each region. B) Radar plots of both clinical phenotypes and atypical subtypes, denoting the mean volume for each region per group. *= *p *≤ 0.05, ** =*p *≤ 0.01, *** =*p *≤ 0.001.

Supplementary Material 3. Supplementary Figure 2. pTau load in the occipital cortex. A) boxplot of pTau load results of the occipital cortex of each group, with examples of high and low immunopositive staining for pTau. B) Radar plot atypical subtypes, denoting the mean volume for each region per group. Note the distinctly higher pTau load in the occipital cortex for the visuospatial group (grey). Hip = hippocampus, ParaHip = parahippocampal gyrus, GFM = middle frontal gyrus, GTM = middle temporal gyrus, GPS = superior parietal gyrus, Precun = precuneus, PCC = posterior cingulate cortex, OC = occipital cortex.

Supplementary Material 4. Supplementary Figure 3. Semi-quantitative amyloid plaque scoring. Scoring (None, very sparse, sparse and frequent) as percentage of scoring observed in a group (control, typical AD or Atypical AD), totaling to 100%. * = *p* ≤ 0.05. Hip = hippocampus, ParaHip = parahippocampal gyrus, GFM = middle frontal gyrus, GTM = middle temporal gyrus, GPS = superior parietal gyrus, Precun = precuneus, PCC = posterior cingulate cortex, OC = occipital cortex

Supplementary Material 5. Supplementary Figure 4. Microvascular vessel area : vessel diameter (ratio) quantification. A) Boxplots of the ratio across all cortical regions of each group, with only the precuneus showing significant differences between control and AD phenotype groups. B) Radarplots of both clinical phenotypes and atypical subtypes, denoting the mean ratio for each region per group. * = *p *≤ 0.05.Hip = hippocampus, ParaHip = parahippocampal gyrus, GFM = middle frontal gyrus, GTM = middle temporal gyrus, GPS = superior parietal gyrus, Precun = precuneus, PCC = posterior cingulate cortex, OC = occipital cortex.

Supplementary Material 6. Supplementary Figure 5. Association between volume and NfL in the middle frontal gyrus. Scatterplot with the linear mixed model derived regression line of hippocampal volume association with COLIV load for the atypical AD group, with its subtypes color coded. Examples of NFL immunoreactivity in the middle frontal gyrus are displayed of relatively low (top) and high (bottom) COLIV load both for the original image (left) and positive signal mask (right).

Supplementary Material 7. Supplementary Figure 6. Association between hippocampal volume and Collagen IV. Scatterplot with the linear mixed model derived regression lines of hippocampal volume associations with COLIV load for each group. Examples of collagen IV immunoreactivity in the hippocampus (specifically in CA4/Subiculum crossover area) in typical AD cases are displayed of relatively low (top) and high (bottom) COLIV load for the original image (left) and positive signal mask (right).

## Data Availability

No datasets were generated or analysed during the current study.

## References

[1] Pini L, Pievani M, Bocchetta M, Altomare D, Bosco P, Cavedo E, et al. Brain atrophy in Alzheimer’s disease and aging. Ageing Res Rev. 2016;30:25–48. 26827786 10.1016/j.arr.2016.01.002

[2] Scheltens P, De Strooper B, Kivipelto M, Holstege H, Chetelat G, Teunissen CE, et al. Alzheimer’s disease. Lancet. 2021;397(10284):1577–90. 33667416 10.1016/S0140-6736(20)32205-4 PMC8354300

[3] Dubois B, Feldman HH, Jacova C, Dekosky ST, Barberger-Gateau P, Cummings J, et al. Research criteria for the diagnosis of Alzheimer’s disease: revising the NINCDS-ADRDA criteria. Lancet Neurol. 2007;6(8):734–46. 17616482 10.1016/S1474-4422(07)70178-3

[4] Whitwell JL, Graff-Radford J, Tosakulwong N, Weigand SD, Machulda MM, Senjem ML, et al. Imaging correlations of tau, amyloid, metabolism, and atrophy in typical and atypical Alzheimer’s disease. Alzheimers Dement. 2018;14(8):1005–14. 29605222 10.1016/j.jalz.2018.02.020 PMC6097955

[5] Graff-Radford J, Yong KXX, Apostolova LG, Bouwman FH, Carrillo M, Dickerson BC, et al. New insights into atypical Alzheimer’s disease in the era of biomarkers. Lancet Neurol. 2021;20(3):222–34. 33609479 10.1016/S1474-4422(20)30440-3 PMC8056394

[6] Ossenkoppele R, Singleton EH, Groot C, Dijkstra AA, Eikelboom WS, Seeley WW, et al. Research criteria for the behavioral variant of Alzheimer disease: a systematic review and meta-analysis. JAMA Neurol. 2022;79(1):48–60. 34870696 10.1001/jamaneurol.2021.4417 PMC8649917

[7] Townley RA, Graff-Radford J, Mantyh WG, Botha H, Polsinelli AJ, Przybelski SA, et al. Progressive dysexecutive syndrome due to Alzheimer’s disease: a description of 55 cases and comparison to other phenotypes. Brain Commun. 2020;2(1):fcaa068. 32671341 10.1093/braincomms/fcaa068 PMC7325839

[8] Crutch SJ, Lehmann M, Schott JM, Rabinovici GD, Rossor MN, Fox NC. Posterior cortical atrophy. Lancet Neurol. 2012;11(2):170–8. 22265212 10.1016/S1474-4422(11)70289-7 PMC3740271

[9] Rogalski E, Sridhar J, Rader B, Martersteck A, Chen K, Cobia D, et al. Aphasic variant of Alzheimer disease: clinical, anatomic, and genetic features. Neurology. 2016;87(13):1337–43. 27566743 10.1212/WNL.0000000000003165 PMC5047036

[10] Ossenkoppele R, Pijnenburg YA, Perry DC, Cohn-Sheehy BI, Scheltens NM, Vogel JW, et al. The behavioural/dysexecutive variant of Alzheimer’s disease: clinical, neuroimaging and pathological features. Brain. 2015;138(Pt 9):2732–49. 26141491 10.1093/brain/awv191 PMC4623840

[11] Sintini I, Graff-Radford J, Schwarz CG, Machulda MM, Singh NA, Carlos AF, et al. Longitudinal rates of atrophy and tau accumulation differ between the visual and language variants of atypical Alzheimer’s disease. Alzheimers Dement. 2023;19(10):4396–406. 37485642 10.1002/alz.13396 PMC10592409

[12] Ossenkoppele R, Schonhaut DR, Scholl M, Lockhart SN, Ayakta N, Baker SL, et al. Tau PET patterns mirror clinical and neuroanatomical variability in Alzheimer’s disease. Brain. 2016;139(Pt 5):1551–67. 26962052 10.1093/brain/aww027 PMC5006248

[13] Therriault J, Pascoal TA, Savard M, Benedet AL, Chamoun M, Tissot C, et al. Topographic distribution of amyloid-beta, Tau, and atrophy in patients with behavioral/dysexecutive Alzheimer disease. Neurology. 2021;96(1):e81–92. 33093220 10.1212/WNL.0000000000011081 PMC7884976

[14] Alobuia WM, Xia W, Vohra BP. Axon degeneration is key component of neuronal death in amyloid-beta toxicity. Neurochem Int. 2013;63(8):782–9. 24083988 10.1016/j.neuint.2013.08.013 PMC3918889

[15] Salvadores N, Geronimo-Olvera C, Court FA. Axonal degeneration in AD: the contribution of abeta and Tau. Front Aging Neurosci. 2020;12: 581767. 33192476 10.3389/fnagi.2020.581767 PMC7593241

[16] Salvadores N, Sanhueza M, Manque P, Court FA. Axonal degeneration during aging and its functional role in neurodegenerative disorders. Front Neurosci. 2017;11: 451. 28928628 10.3389/fnins.2017.00451 PMC5591337

[17] Svenningsson AL, Stomrud E, Palmqvist S, Hansson O, Ossenkoppele R. Axonal degeneration and amyloid pathology predict cognitive decline beyond cortical atrophy. Alzheimers Res Ther. 2022;14(1):144. 36192766 10.1186/s13195-022-01081-w PMC9531524

[18] Berth SH, Lloyd TE. Disruption of axonal transport in neurodegeneration. J Clin Invest. 2023;133(11):e168554. 37259916 10.1172/JCI168554 PMC10232001

[19] Khalil M, Teunissen CE, Otto M, Piehl F, Sormani MP, Gattringer T, et al. Neurofilaments as biomarkers in neurological disorders. Nat Rev Neurol. 2018;14(10):577–89. 30171200 10.1038/s41582-018-0058-z

[20] Lee S, Eyer J, Letournel F, Boumil E, Hall G, Shea TB. Neurofilaments form flexible bundles during neuritogenesis in culture and in mature axons in situ. J Neurosci Res. 2019;97(10):1306–18. 31304612 10.1002/jnr.24482

[21] Ashton NJ, Janelidze S, Al Khleifat A, Leuzy A, van der Ende EL, Karikari TK, et al. A multicentre validation study of the diagnostic value of plasma neurofilament light. Nat Commun. 2021;12(1):3400. 34099648 10.1038/s41467-021-23620-z PMC8185001

[22] Kang MS, Aliaga AA, Shin M, Mathotaarachchi S, Benedet AL, Pascoal TA, et al. Amyloid-beta modulates the association between neurofilament light chain and brain atrophy in Alzheimer’s disease. Mol Psychiatry. 2021;26(10):5989–6001. 32591633 10.1038/s41380-020-0818-1 PMC8758474

[23] Dhiman K, Gupta VB, Villemagne VL, Eratne D, Graham PL, Fowler C, et al. Cerebrospinal fluid neurofilament light concentration predicts brain atrophy and cognition in Alzheimer’s disease. Alzheimers Dement (Amst). 2020;12(1): e12005. 32211500 10.1002/dad2.12005 PMC7085283

[24] Paterson RW, Toombs J, Slattery CF, Nicholas JM, Andreasson U, Magdalinou NK, et al. Dissecting IWG-2 typical and atypical Alzheimer’s disease: insights from cerebrospinal fluid analysis. J Neurol. 2015;262(12):2722–30. 26410752 10.1007/s00415-015-7904-3

[25] Greenberg SM, Bacskai BJ, Hernandez-Guillamon M, Pruzin J, Sperling R, van Veluw SJ. Cerebral amyloid angiopathy and Alzheimer disease - one peptide, two pathways. Nat Rev Neurol. 2020;16(1):30–42. 31827267 10.1038/s41582-019-0281-2 PMC7268202

[26] Steinman J, Sun HS, Feng ZP. Microvascular Alterations in Alzheimer’s Disease. Front Cell Neurosci. 2020;14:618986. 33536876 10.3389/fncel.2020.618986 PMC7849053

[27] Richard E, van Gool WA, Hoozemans JJ, van Haastert ES, Eikelenboom P, Rozemuller AJ, et al. Morphometric changes in the cortical microvascular network in Alzheimer’s disease. J Alzheimers Dis. 2010;22(3):811–8. 20858968 10.3233/JAD-2010-100849

[28] Hunter JM, Kwan J, Malek-Ahmadi M, Maarouf CL, Kokjohn TA, Belden C, et al. Morphological and pathological evolution of the brain microcirculation in aging and Alzheimer’s disease. PLoS ONE. 2012;7(5): e36893. 22615835 10.1371/journal.pone.0036893 PMC3353981

[29] van der Flier WM, Scheltens P. Amsterdam dementia cohort: performing research to optimize care. J Alzheimers Dis. 2018;62(3):1091–111. 29562540 10.3233/JAD-170850 PMC5870023

[30] van der Flier WM, Pijnenburg YA, Prins N, Lemstra AW, Bouwman FH, Teunissen CE, et al. Optimizing patient care and research: the Amsterdam Dementia Cohort. J Alzheimers Dis. 2014;41(1):313–27. 24614907 10.3233/JAD-132306

[31] Alafuzoff I, Parkkinen L, Al-Sarraj S, Arzberger T, Bell J, Bodi I, et al. Assessment of alpha-synuclein pathology: a study of the BrainNet Europe Consortium. J Neuropathol Exp Neurol. 2008;67(2):125–43. 18219257 10.1097/nen.0b013e3181633526

[32] Alafuzoff I, Thal DR, Arzberger T, Bogdanovic N, Al-Sarraj S, Bodi I, et al. Assessment of beta-amyloid deposits in human brain: a study of the BrainNet Europe Consortium. Acta Neuropathol. 2009;117(3):309–20. 19184666 10.1007/s00401-009-0485-4 PMC2910889

[33] Jonkman LE, Graaf YG, Bulk M, Kaaij E, Pouwels PJW, Barkhof F, et al. Normal Aging Brain Collection Amsterdam (NABCA): A comprehensive collection of postmortem high-field imaging, neuropathological and morphometric datasets of non-neurological controls. Neuroimage Clin. 2019;22: 101698. 30711684 10.1016/j.nicl.2019.101698 PMC6360607

[34] Cavedo E, Tran P, Thoprakarn U, Martini JB, Movschin A, Delmaire C, et al. Validation of an automatic tool for the rapid measurement of brain atrophy and white matter hyperintensity: QyScore(R). Eur Radiol. 2022;32(5):2949–61. 34973104 10.1007/s00330-021-08385-9 PMC9038894

[35] Tran P, Thoprakarn U, Gourieux E, Dos Santos CL, Cavedo E, Guizard N, et al. Automatic segmentation of white matter hyperintensities: validation and comparison with state-of-the-art methods on both Multiple Sclerosis and elderly subjects. Neuroimage Clin. 2022;33: 102940. 35051744 10.1016/j.nicl.2022.102940 PMC8896108

[36] Chupin M, Hammers A, Liu RS, Colliot O, Burdett J, Bardinet E, et al. Automatic segmentation of the hippocampus and the amygdala driven by hybrid constraints: method and validation. Neuroimage. 2009;46(3):749–61. 19236922 10.1016/j.neuroimage.2009.02.013 PMC2677639

[37] Rolls ET, Huang CC, Lin CP, Feng J, Joliot M. Automated anatomical labelling atlas 3. Neuroimage. 2020;206: 116189. 31521825 10.1016/j.neuroimage.2019.116189

[38] Arendt T, Morawski M, Gartner U, Frohlich N, Schulze F, Wohmann N, et al. Inhomogeneous distribution of Alzheimer pathology along the isocortical relief. Are cortical convolutions an Achilles heel of evolution? Brain Pathol. 2017;27(5):603–11. 27564538 10.1111/bpa.12442 PMC8029161

[39] Adler DH, Pluta J, Kadivar S, Craige C, Gee JC, Avants BB, et al. Histology-derived volumetric annotation of the human hippocampal subfields in postmortem MRI. Neuroimage. 2014;84:505–23. 24036353 10.1016/j.neuroimage.2013.08.067 PMC3864597

[40] Insausti R, Munoz-Lopez M, Insausti AM, Artacho-Perula E. The human periallocortex: layer pattern in presubiculum, parasubiculum and entorhinal cortex. A Review Front Neuroanat. 2017;11:84. 29046628 10.3389/fnana.2017.00084 PMC5632821

[41] Schindelin J, Arganda-Carreras I, Frise E, Kaynig V, Longair M, Pietzsch T, et al. Fiji: an open-source platform for biological-image analysis. Nat Methods. 2012;9(7):676–82. 22743772 10.1038/nmeth.2019 PMC3855844

[42] Thal DR, Rub U, Schultz C, Sassin I, Ghebremedhin E, Del Tredici K, et al. Sequence of Abeta-protein deposition in the human medial temporal lobe. J Neuropathol Exp Neurol. 2000;59(8):733–48. 10952063 10.1093/jnen/59.8.733

[43] Boon BDC, Bulk M, Jonker AJ, Morrema THJ, van den Berg E, Popovic M, et al. The coarse-grained plaque: a divergent Abeta plaque-type in early-onset Alzheimer’s disease. Acta Neuropathol. 2020;140(6):811–30. 32926214 10.1007/s00401-020-02198-8 PMC7666300

[44] Mirra SS, Heyman A, McKeel D, Sumi SM, Crain BJ, Brownlee LM, et al. The Consortium to Establish a Registry for Alzheimer’s Disease (CERAD). Part II. Standardization of the neuropathologic assessment of Alzheimer’s disease. Neurology. 1991;41(4):479–86. 2011243 10.1212/wnl.41.4.479

[45] Koo TK, Li MY. A Guideline of Selecting and Reporting Intraclass Correlation Coefficients for Reliability Research. J Chiropr Med. 2016;15(2):155–63. 27330520 10.1016/j.jcm.2016.02.012 PMC4913118

[46] Whitwell JL. Progression of atrophy in Alzheimer’s disease and related disorders. Neurotox Res. 2010;18(3–4):339–46. 20352396 10.1007/s12640-010-9175-1

[47] Planche V, Manjon JV, Mansencal B, Lanuza E, Tourdias T, Catheline G, et al. Structural progression of Alzheimer’s disease over decades: the MRI staging scheme. Brain Commun. 2022;4(3):fcac109. 35592489 10.1093/braincomms/fcac109 PMC9113086

[48] Farzan A, Mashohor S, Ramli R, Mahmud R. Discriminant analysis of intermediate brain atrophy rates in longitudinal diagnosis of Alzheimer’s disease. Diagn Pathol. 2011;6:105. 22035255 10.1186/1746-1596-6-105 PMC3305898

[49] Mutlu J, Landeau B, Tomadesso C, de Flores R, Mezenge F, de La Sayette V, et al. Connectivity Disruption, Atrophy, and Hypometabolism within Posterior Cingulate Networks in Alzheimer’s Disease. Front Neurosci. 2016;10:582. 28066167 10.3389/fnins.2016.00582 PMC5174151

[50] Pengas G, Hodges JR, Watson P, Nestor PJ. Focal posterior cingulate atrophy in incipient Alzheimer’s disease. Neurobiol Aging. 2010;31(1):25–33. 18455838 10.1016/j.neurobiolaging.2008.03.014

[51] Buckner RL, Snyder AZ, Shannon BJ, LaRossa G, Sachs R, Fotenos AF, et al. Molecular, structural, and functional characterization of Alzheimer’s disease: evidence for a relationship between default activity, amyloid, and memory. J Neurosci. 2005;25(34):7709–17. 16120771 10.1523/JNEUROSCI.2177-05.2005 PMC6725245

[52] Vogel JW, Corriveau-Lecavalier N, Franzmeier N, Pereira JB, Brown JA, Maass A, et al. Connectome-based modelling of neurodegenerative diseases: towards precision medicine and mechanistic insight. Nat Rev Neurosci. 2023;24(10):620–39. 37620599 10.1038/s41583-023-00731-8

[53] Pini L, Wennberg AM, Salvalaggio A, Vallesi A, Pievani M, Corbetta M. Breakdown of specific functional brain networks in clinical variants of Alzheimer’s disease. Ageing Res Rev. 2021;72:101482. 34606986 10.1016/j.arr.2021.101482

[54] Steketee RM, Bron EE, Meijboom R, Houston GC, Klein S, Mutsaerts HJ, et al. Early-stage differentiation between presenile Alzheimer’s disease and frontotemporal dementia using arterial spin labeling MRI. Eur Radiol. 2016;26(1):244–53. 26024845 10.1007/s00330-015-3789-x PMC4666273

[55] Boxer AL, Rankin KP, Miller BL, Schuff N, Weiner M, Gorno-Tempini ML, et al. Cinguloparietal atrophy distinguishes Alzheimer disease from semantic dementia. Arch Neurol. 2003;60(7):949–56. 12873851 10.1001/archneur.60.7.949

[56] Pegueroles J, Vilaplana E, Montal V, Sampedro F, Alcolea D, Carmona-Iragui M, et al. Longitudinal brain structural changes in preclinical Alzheimer’s disease. Alzheimers Dement. 2017;13(5):499–509. 27693189 10.1016/j.jalz.2016.08.010

[57] Fortea J, Vilaplana E, Alcolea D, Carmona-Iragui M, Sanchez-Saudinos MB, Sala I, et al. Cerebrospinal fluid beta-amyloid and phospho-tau biomarker interactions affecting brain structure in preclinical Alzheimer disease. Ann Neurol. 2014;76(2):223–30. 24852682 10.1002/ana.24186

[58] Johnson SC, Christian BT, Okonkwo OC, Oh JM, Harding S, Xu G, et al. Amyloid burden and neural function in people at risk for Alzheimer’s Disease. Neurobiol Aging. 2014;35(3):576–84. 24269021 10.1016/j.neurobiolaging.2013.09.028 PMC4018215

[59] Ingala S, De Boer C, Masselink LA, Vergari I, Lorenzini L, Blennow K, et al. Application of the ATN classification scheme in a population without dementia: Findings from the EPAD cohort. Alzheimers Dement. 2021;17(7):1189–204. 33811742 10.1002/alz.12292 PMC8359976

[60] Williams ME, Elman JA, Bell TR, Dale AM, Eyler LT, Fennema-Notestine C, et al. Higher cortical thickness/volume in Alzheimer’s-related regions: protective factor or risk factor? Neurobiol Aging. 2023;129:185–94. 37343448 10.1016/j.neurobiolaging.2023.05.004 PMC10676195

[61] Frigerio I, Boon BDC, Lin CP, Galis-de Graaf Y, Bol J, Preziosa P, et al. Amyloid-beta, p-tau and reactive microglia are pathological correlates of MRI cortical atrophy in Alzheimer’s disease. Brain Commun. 2021;3(4):fcab281. 34927073 10.1093/braincomms/fcab281 PMC8677327

[62] Alves F, Kalinowski P, Ayton S. Accelerated Brain Volume Loss Caused by Anti-beta-Amyloid Drugs: A Systematic Review and Meta-analysis. Neurology. 2023;100(20):e2114–24. 36973044 10.1212/WNL.0000000000207156 PMC10186239

[63] Barkhof F, Knopman DS. Brain Shrinkage in Anti-beta-Amyloid Alzheimer Trials: Neurodegeneration or Pseudoatrophy? Neurology. 2023;100(20):941–2. 36973045 10.1212/WNL.0000000000207268

[64] Belder CRS, Boche D, Nicoll JAR, Jaunmuktane Z, Zetterberg H, Schott JM, et al. Brain volume change following anti-amyloid beta immunotherapy for Alzheimer’s disease: amyloid-removal-related pseudo-atrophy. Lancet Neurol. 2024;23(10):1025–34. 39304242 10.1016/S1474-4422(24)00335-1

[65] Das SR, Lyu X, Duong MT, Xie L, McCollum L, de Flores R, et al. Tau-Atrophy Variability Reveals Phenotypic Heterogeneity in Alzheimer’s Disease. Ann Neurol. 2021;90(5):751–62. 34617306 10.1002/ana.26233 PMC8841129

[66] Palmqvist S, Eshaghi A. Spatial Distribution of Tau and beta-Amyloid Pathologies and Their Role in Different Alzheimer Disease Phenotypes. Neurology. 2021;96(5):191–2. 33262229 10.1212/WNL.0000000000011272

[67] Carlos AF, Tosakulwong N, Weigand SD, Buciuc M, Ali F, Clark HM, et al. Histologic lesion type correlates of magnetic resonance imaging biomarkers in four-repeat tauopathies. Brain Commun. 2022;4(3):fcac108. 35663380 10.1093/braincomms/fcac108 PMC9155234

[68] Moloney CM, Lowe VJ, Murray ME. Visualization of neurofibrillary tangle maturity in Alzheimer’s disease: A clinicopathologic perspective for biomarker research. Alzheimers Dement. 2021;17(9):1554–74. 33797838 10.1002/alz.12321 PMC8478697

[69] Ravikumar S, Denning AE, Lim S, Chung E, Sadeghpour N, Ittyerah R, et al. Postmortem imaging reveals patterns of medial temporal lobe vulnerability to tau pathology in Alzheimer’s disease. Nat Commun. 2024;15(1):4803. 38839876 10.1038/s41467-024-49205-0 PMC11153494

[70] Moloney CM, Labuzan SA, Crook JE, Siddiqui H, Castanedes-Casey M, Lachner C, et al. Phosphorylated tau sites that are elevated in Alzheimer’s disease fluid biomarkers are visualized in early neurofibrillary tangle maturity levels in the post mortem brain. Alzheimers Dement. 2023;19(3):1029–40. 35920592 10.1002/alz.12749 PMC9895127

[71] Opfer R, Ostwaldt AC, Walker-Egger C, Manogaran P, Sormani MP, De Stefano N, et al. Within-patient fluctuation of brain volume estimates from short-term repeated MRI measurements using SIENA/FSL. J Neurol. 2018;265(5):1158–65. 29549466 10.1007/s00415-018-8825-8

[72] Jefferies WA, Price KA, Biron KE, Fenninger F, Pfeifer CG, Dickstein DL. Adjusting the compass: new insights into the role of angiogenesis in Alzheimer’s disease. Alzheimers Res Ther. 2013;5(6):64. 24351529 10.1186/alzrt230 PMC4056615

[73] Desai BS, Schneider JA, Li JL, Carvey PM, Hendey B. Evidence of angiogenic vessels in Alzheimer’s disease. J Neural Transm (Vienna). 2009;116(5):587–97. 19370387 10.1007/s00702-009-0226-9 PMC2753398

[74] Vagnucci AH Jr, Li WW. Alzheimer’s disease and angiogenesis. Lancet. 2003;361(9357):605–8. 12598159 10.1016/S0140-6736(03)12521-4

[75] Boon BDC, Pouwels PJW, Jonkman LE, Keijzer MJ, Preziosa P, van de Berg WDJ, et al. Can post-mortem MRI be used as a proxy for in vivo? A case study. Brain Commun. 2019;1(1):fcz030. 32954270 10.1093/braincomms/fcz030 PMC7425311

[76] Singh NA, Graff-Radford J, Machulda MM, Carlos AF, Schwarz CG, Senjem ML, et al. Atypical Alzheimer’s disease: new insights into an overlapping spectrum between the language and visual variants. J Neurol. 2024;271(6):3571–85. 38551740 10.1007/s00415-024-12297-1 PMC11273322

[77] Whitwell JL, Martin PR, Graff-Radford J, Machulda MM, Sintini I, Buciuc M, et al. Investigating Heterogeneity and Neuroanatomic Correlates of Longitudinal Clinical Decline in Atypical Alzheimer Disease. Neurology. 2022;98(24):e2436–45. 35483899 10.1212/WNL.0000000000200336 PMC9231842

[78] Boon BDC, Frigerio I, de Gooijer D, Morrema THJ, Bol J, Galis-de Graaf Y, et al. Alzheimer’s disease clinical variants show distinct neuroinflammatory profiles with neuropathology. Neuropathol Appl Neurobiol. 2024;50(5):e13009. 39400356 10.1111/nan.13009 PMC12465012

[79] Mecca AP, O’Dell RS, Sharp ES, Banks ER, Bartlett HH, Zhao W, et al. Synaptic density and cognitive performance in Alzheimer’s disease: A PET imaging study with [(11) C]UCB-J. Alzheimers Dement. 2022;18(12):2527–36. 35174954 10.1002/alz.12582 PMC9381645

[80] Taddei RN, Sanchez-Mico MV, Bonnar O, Connors T, Gaona A, Denbow D, et al. Changes in glial cell phenotypes precede overt neurofibrillary tangle formation, correlate with markers of cortical cell damage, and predict cognitive status of individuals at Braak III-IV stages. Acta Neuropathol Commun. 2022;10(1):72. 35534858 10.1186/s40478-022-01370-3 PMC9082857

[81] Mathys H, Boix CA, Akay LA, Xia Z, Davila-Velderrain J, Ng AP, et al. Single-cell multiregion dissection of Alzheimer’s disease. Nature. 2024;632(8026):858–68. 39048816 10.1038/s41586-024-07606-7 PMC11338834

[82] Glasser MF, Van Essen DC. Mapping human cortical areas in vivo based on myelin content as revealed by T1- and T2-weighted MRI. J Neurosci. 2011;31(32):11597–616. 21832190 10.1523/JNEUROSCI.2180-11.2011 PMC3167149

[83] Pelkmans W, Dicks E, Barkhof F, Vrenken H, Scheltens P, van der Flier WM, et al. Gray matter T1-w/T2-w ratios are higher in Alzheimer’s disease. Hum Brain Mapp. 2019;40(13):3900–9. 31157938 10.1002/hbm.24638 PMC6771703

[84] Weston PSJ, Poole T, Nicholas JM, Toussaint N, Simpson IJA, Modat M, et al. Measuring cortical mean diffusivity to assess early microstructural cortical change in presymptomatic familial Alzheimer’s disease. Alzheimers Res Ther. 2020;12(1):112. 32943095 10.1186/s13195-020-00679-2 PMC7499910

[85] Frisoni GB, Altomare D, Thal DR, Ribaldi F, van der Kant R, Ossenkoppele R, et al. The probabilistic model of Alzheimer disease: the amyloid hypothesis revised. Nat Rev Neurosci. 2022;23(1):53–66. 34815562 10.1038/s41583-021-00533-w PMC8840505

