# Supplementary material for: Clinical phenotypes of Alzheimer’s disease: investigating atrophy patterns and their pathological correlates
Source: Alzheimers Res Ther. 2025 Apr 26;17:93. doi: 10.1186/s13195-025-01727-5 (PMC12032798; doi:10.1186/s13195-025-01727-5)
Supplement: Supplementary file 1 — Supplementary Material 1. Supplementary Table 1. Ante-mortem MRI scan specifications. Supplementary Table 2. Cohort characteristics per case. Supplementary Table 3. Cortical Volume and Pathological load per Phenotype group and subtypes. Supplementary table 4. Significant results from group comparisons in regional volume of AAL3 regions. Supplementary Table 5. Correlation analysis between MRI volume measurements and visual atrophy scores. Supplementary Table 6. Correlation analysis between pathologies. Supplementary Table 7. Correlation analysis between CAA and Aβ load, COLIV load and ratio. Supplementary Table 8. Linear mixed model results regional volume pathology associations. [file 13195_2025_1727_MOESM1_ESM.docx]

**Supplementary Table 1. Ante-mortem MRI scan specifications**

| Case | Date | Years before death | Scanner | Dimensions | In-plane resolution | TE | TR |
| --- | --- | --- | --- | --- | --- | --- | --- |
| 4 | 2011 | 5 | GE3T0 Signa HDxt | 176x256x256 | 1 | 3.2 | 7.8 |
| 4 | 2014 | 2 | GE3T0 Discovery MR750 | 176x256x256 | 1 | 3.2 | 8.2 |
| 5 | 2009 | 7 | GE3T0 Signa HDxt | 176x256x256 | 1 | 3.2 | 7.8 |
| 6 | 2015a | 2 | Philips Ingenuity | 192x256x256 | 1 | 4.5 | 7.9 |
| 6 | 2015b | 2 | Philips Ingenuity | 192x256x256 | 1 | 4.5 | 7.9 |
| 9 | 2010 | 7 | GE3T0 Signa HDxt | 176x256x256 | 1 | 3.2 | 7.8 |
| 9 | 2015 | 2 | TOSHIBA Titan3T | 176x256x256 | 1 | 3.4 | 7.2 |
| 10 | 2012 | 5 | GE3T0 Signa HDxt | 176x256x256 | 1 | 3 | 7.8 |
| 10 | 2014 | 3 | TOSHIBA Titan3T | 176x256x256 | 1 | 3.2 | 9.5 |
| 11 | 2017 | <1 | GE3T0 Discovery MR750 | 176x256x256 | 1 | 2.9 | 6.7 |
| 13 | 2011 | 7 | GE3T0 Signa HDxt | 176x256x256 | 1 | 3 | 7.8 |
| 13 | 2017 | 1 | Philips Ingenuity | 192x256x256 | 1 | 4.5 | 7.9 |
| 15 | 2013 | 5 | GE3T0 Discovery MR750 | 176x256x256 | 1 | 3.2 | 8.2 |
| 16 | 2018 | <1 | Philips Ingenuity | 192x256x256 | 1 | 4.5 | 7.9 |
| 17 | 2014 | 4 | TOSHIBA Titan3T | 176x256x256 | 1 | 3.2 | 9.5 |
| 18 | 2018 | <1 | SIEMENS Avanto | 160x256x192 | 1 | 5.2 | 2700 |
| 19 | 2016 | 2 | GE3T0 Discovery MR750 | 176x256x256 | 1 | 3.2 | 8.2 |
| 20 | 2014 | 5 | TOSHIBA Titan3T | 176x256x256 | 1 | 3.2 | 9.5 |
| 21 | 2011 | 8 | GE3T0 Signa HDxt | 176x256x256 | 1 | 3 | 7.8 |
| 21 | 2013 | 6 | Philips Ingenuity | 192x256x256 | 1 | 3 | 7 |
| 22 | 2016 | 3 | Philips Ingenuity | 192x256x256 | 1 | 4.5 | 7.9 |
| 22 | 2014a | 5 | TOSHIBA Titan3T | 176x256x256 | 1 | 3.2 | 9.5 |
| 22 | 2014b | 5 | Philips Ingenuity | 192x256x256 | 1 | 4.5 | 7.9 |
| 23 | 2016a | 3 | TOSHIBA Titan3T | 176x256x256 | 1 | 2.4 | 5.7 |
| 23 | 2016b | 3 | GE3T0 Discovery MR750 | 176x256x256 | 1 | 3.2 | 8.2 |
| 23 | 2017a | 2 | GE3T0 Discovery MR750 | 176x256x256 | 1 | 3.2 | 8.2 |
| 23 | 2017b | 2 | Philips Ingenuity | 192x256x256 | 1 | 4.5 | 7.9 |
| 23 | 2019 | <1 | Philips Ingenuity | 192x256x256 | 1 | 4.5 | 7.9 |
| 25 | 2012 | 7 | GE3T0 Signa HDxt | 176x256x256 | 1 | 3 | 7.8 |
| 26 | 2014 | 5 | TOSHIBA Titan3T | 176x256x256 | 1 | 3.2 | 9.5 |
| 28 | 2018 | 1 | Philips Ingenuity | 192x256x256 | 1 | 4.5 | 7.9 |
| 29 | 2015 | 4 | GE3T0 Discovery MR750 | 176x256x256 | 1 | 3.2 | 8.2 |
| 30 | 2010 | 10 | SIEMENS Sonata | 198x256x256 | 1 | 5.1 | 2400 |
| 30 | 2013 | 7 | GE1T5 Signa HDxt | 180x256x256 | 1.2 | 5.1 | 12.4 |
| 30 | 2014 | 6 | GE3T0 Discovery MR750 | 176x256x256 | 1 | 3.2 | 8.2 |
| 30 | 2011a | 9 | SIEMENS Sonata | 198x256x256 | 1 | 5.1 | 2400 |
| 30 | 2011b | 9 | GE3T0 Signa HDxt | 176x256x256 | 1 | 3 | 7.8 |
| 30 | 2011c | 9 | GE3T0 Signa HDxt | 176x256x256 | 1 | 3 | 7.8 |
| 30 | 2012a | 8 | SIEMENS Sonata | 198x256x256 | 1 | 5.1 | 2400 |
| 30 | 2012b | 8 | GE3T0 Signa HDxt | 176x256x256 | 1 | 3 | 7.8 |
| 32 | 2018 | 3 | TOSHIBA Titan3T | 176x256x256 | 1.2 | 2.4 | 5.7 |
| 34 | 2017 | 5 | Philips Ingenuity | 192x256x256 | 1 | 4.5 | 7.9 |
| 34 | 2019 | 3 | Philips Ingenuity | 192x256x256 | 1 | 4.5 | 7.9 |
| 35 | 2012 | 10 | GE3T0 Signa HDxt | 176x256x256 | 1 | 3 | 7.8 |
| 37 | 2014 | 8 | TOSHIBA Titan3T | 176x256x256 | 1 | 3.2 | 9.5 |
| 37 | 2017 | 5 | Philips Ingenuity | 192x256x256 | 1 | 4.5 | 7.9 |
| 39 | 2021 | 1 | Siemens MAGNETOM vida | 192x256x256 | 0.9 | 2.32 | 2300 |
| Case numbers match numbers in supplementary table 2. All cases are diagnosed AD cases. Letters a or b behind scan date signifies first or second scan of the given date. TE = echo time, TR = repetition time. | | | | | | | |

**Supplementary Table 2. Cohort characteristics per case.**

| **Case code** | **Subtype** | **Sex** | **Age at death** | **Cause of death** | **PMD** | **Disease duration** | **CDR** | **APOE** | **Braak NFT** | **Thal** | **CAA-Type** | **Braak LB** | **GCA** | **PCA** | **MTA** | **Fazekas** | **NWBV** | **NGMV** | **NWMV** |  |
| --- | --- | --- | --- | --- | --- | --- | --- | --- | --- | --- | --- | --- | --- | --- | --- | --- | --- | --- | --- | --- |
| **Typical AD** |  |  |  |  |  |  |  |  |  |  |  |  |  |  |  |  |  |  |  |  |
| AD 02 | Typical | M | 60 | Euthanasia | 08:35 | 2 | NA | 3/3 | 6 | 5 | 1 | 0 | 0 | 1 | 0 | 3 | 67.79 | 36.85 | 30.93 |  |
| AD 04 | Typical | M | 68 | Euthanasia | 09:15 | 6 | NA | 3/3 | 5 | 5 | 2 | 0 | 2 | 1 | 1 | 1 | 70.40 | 40.48 | 29.92 |  |
| AD 05 | Typical | M | 69 | Pneumonia | 11:55 | 11 | 3 | 3/4 | 5 | 5 | 1 | 0 | 1 | 2 | 3 | 0 | 59.72 | 37.22 | 22.50 |  |
| AD 09 | Typical | M | 84 | Euthanasia | 05:53 | 13 | 1 | 3/4 | 4 | 5 | 1 | 0 | 1 | 2 | 3 | 1 | 62.51 | 32.04 | 30.47 |  |
| AD 13 | Typical | F | 80 | Euthanasia | 07:05 | 7 | 1 | 3/3 | 4 | 5 | 1 | 0 | 1 | 1 | 2 | 1 | 67.17 | 40.53 | 26.64 |  |
| AD 17 | Typical | M | 53 | End-Stage Disease | 09:00 | 5 | 3 | 3/3 | 6 | 5 | 1 | 0 | 3 | 2 | 1 | 0 | 54.87 | 31.18 | 23.69 |  |
| AD 18 | Typical | M | 64 | Cachexia | 07:55 | 12 | 3 | 3/4 | 6 | 5 | 2 | 0 | 2 | 1 | 4 | 0 | 52.04 | 29.71 | 22.33 |  |
| AD 21 | Typical | M | 84 | Euthanasia | 08:35 | 14 | NA | 3/3 | 4 | 3 | 0 | 0 | 2 | 1 | 2 | 2 | 58.82 | 36.29 | 22.53 |  |
| AD 22 | Typical | M | 77 | Suicide by drugs | 09:05 | 10 | NA | 4/4 | 6 | 5 | 1 | 0 | 1 | 1 | 1 | 3 | 63.68 | 35.51 | 28.17 |  |
| AD 23 | Typical | M | 65 | Euthanasia | 09:20 | 7 | 1 | 3/4 | 5 | 5 | 1 | 0 | 0 | 1 | 1 | 1 | 67.22 | 40.37 | 26.84 |  |
| AD 25 | Typical | M | 63 | Myocardial infarction | 08:45 | 10 | 2 | 3/4 | 6 | 5 | 1 | 0 | 1 | 3 | 1 | 0 | 59.03 | 36.01 | 23.02 |  |
| AD 30 | Typical | F | 61 | End-Stage Disease | 07:40 | 6 | NA | 4/4 | 6 | 5 | 1 | 0 | 1 | 3 | 3 | 0 | 60.20 | 37.91 | 22.28 |  |
| AD 31 | Typical | F | 53 | End-Stage Disease | 06:30 | 7 | 2 | 2/3 | 6 | 5 | 1 | 0 | 1 | 1 | 2 | 3 | 62.21 | 31.30 | 30.92 |  |
| AD 32 | Typical | M | 60 | Euthanasia | 07:05 | 2 | 1 | 4/4 | 6 | 5 | 1 | 0 | 1 | 2 | 2 | 1 | 64.15 | 38.07 | 26.08 |  |
| AD 35 | Typical | M | 67 | Euthanasia | 08:00 | 10 | 1 | 3/4 | 6 | 5 | 1 | 4 | 1 | 2 | 3 | 0 | 55.35 | 35.34 | 20.01 |  |
| AD 37 | Typical | M | 79 | Unknown | 08:05 | 8 | NA | 3/3 | 5 | 5 | 1 | 2 | 2 | 1 | 4 | 3 | 50.42 | 35.33 | 15.09 |  |
| AD 38 | Typical | F | 89 | End-Stage Disease | 06:35 | 8 | 3 | 4/4 | 5 | 5 | 1 | 5 | 1 | 2 | 4 | 3 | 60.06 | 36.12 | 23.94 |  |
| **Atypical AD** |  |  |  |  | 07:00 |  |  |  |  |  |  |  |  |  |  |  |  |  |  |  |
| AD 16 | Behavioural | M | 77 | Euthanasia | 07:20 | 4 | 1 | 3/4 | 4 | 5 | 2 | 0 | 0 | 0 | 0 | 1 | 70.24 | 39.58 | 30.66 |  |
| AD 20 | Behavioural | M | 73 | Cachexia | 06:15 | 10 | 3 | 3/4 | 5 | 5 | 1 | 5 | 3 | 3 | 3 | 2 | 52.03 | 25.45 | 26.59 |  |
| AD 27 | Behavioural | F | 73 | Pneumothorax | 06:30 | 1 | 3 | 3/4 | 6 | 5 | 2 | 0 | 0 | 1 | 3 | 0 | 59.71 | 38.32 | 21.38 |  |
| AD 07 | Dysexecutive | M | 59 | Euthanasia | 07:30 | 2 | 2 | 4/4 | 5 | 5 | 1 | 5 | 0 | 2 | 1 | 2 | 73.25 | 42.20 | 31.05 |  |
| AD 08 | Dysexecutive | F | 78 | Cachexia | 11:11 | 4 | 3 | 3/4 | 5 | 5 | 1 | 3 | 1 | 2 | 3 | 3 | 63.89 | 35.66 | 28.22 |  |
| AD 11 | Dysexecutive | M | 37 | Euthanasia | 08:55 | 5 | 1 | 2/3 | 6 | 5 | 1 | 0 | 0 | 1 | 1 | 0 | 71.99 | 41.83 | 30.17 |  |
| AD 12 | Dysexecutive | M | 58 | Cachexia | 05:35 | 2 | 3 | 3/4 | 6 | 5 | 1 | 0 | 1 | 2 | 1 | 0 | 64.93 | 36.84 | 28.09 |  |
| AD 19 | Dysexecutive | M | 59 | Cachexia | 05:05 | 3 | 3 | 3/4 | 5 | 5 | 1 | 0 | 1 | 2 | 3 | 2 | 52.23 | 30.67 | 21.56 |  |
| AD 26 | Logopenic | F | 72 | End-Stage Disease | 09:45 | 5 | 1 | 2/3 | 6 | 5 | 2 | 0 | 2 | 2 | 2 | 2 | 51.79 | 26.07 | 25.72 |  |
| AD 34 | Logopenic | M | 75 | End-Stage Disease | 08:15 | 5 | 3 | 4/4 | 6 | 5 | 1 | 0 | 3 | 2 | 4 | 3 | 58.08 | 32.47 | 25.61 |  |
| AD 39 | Logopenic | F | 67 | End-Stage Disease | 08:15 | 1 | 3 | 3/3 | 5 | 5 | 1 | 0 | 1 | 2 | 2 | 1 | 58.67 | 32.99 | 25.69 |  |
| AD 10 | Visuospatial | M | 62 | End-Stage Disease | 06:35 | 5 | 3 | 3/4 | 6 | 4 | 1 | 0 | 2 | 3 | 2 | 3 | 51.93 | 28.03 | 23.91 |  |
| AD 15 | Visuospatial | M | 67 | Cachexia | 10:50 | 9 | 3 | 3/4 | 6 | 5 | 2 | 0 | 3 | 2 | 2 | 0 | 55.56 | 31.36 | 24.20 |  |
| AD 28 | Visuospatial | F | 60 | Euthanasia | 06:22 | 2 | 2 | 3/3 | 6 | 5 | 2 | 0 | 1 | 3 | 1 | 1 | 66.98 | 39.78 | 27.19 |  |
| AD 29 | Visuospatial | M | 68 | End-Stage Disease | 07:50 | 3 | NA | 3/4 | 6 | 5 | 2 | 0 | 1 | 1 | 3 | 2 | 56.36 | 39.88 | 16.48 |  |
| AD 06 | NA | M | 65 | Cardiac arrest | 08:40 | 7 | NA | 3/3 | 5 | 4 | 1 | 0 | 0 | 1 | 1 | 2 | 69.62 | 37.43 | 32.19 |  |
| **Control** |  |  |  |  | 08:00 |  |  |  |  |  |  |  |  |  |  |  |  |  |  |  |
| Control 1 | Control | M | 68 | Euthanasia | 10:30 | - | - | 3/4 | 1 | 2 | 2 | 0 | 0 | 0 | 0 | 0 | 75.88 | 45.24 | 30.64 |  |
| Control 2 | Control | F | 63 | Euthanasia | 09:20 | - | - | 2/3 | 0 | 0 | 0 | 0 | 0 | 0 | 0 | 0 | 76.38 | 42.94 | 33.43 |  |
| Control 3 | Control | M | 82 | Intestinal perforation | 08:25 | - | - | 2/3 | 1 | 1 | 0 | 0 | 0 | 1 | 0 | 2 | 68.25 | 37.83 | 30.42 |  |
| Control 4 | Control | M | 85 | Euthanasia | 07:30 | - | - | 3/3 | 1 | 1 | 0 | 0 | 0 | 0 | 1 | 2 | 66.80 | 36.16 | 30.64 |  |
| Control 5 | Control | M | 67 | Euthanasia | 08:35 | - | - | 3/4 | 2 | 1 | 0 | 0 | 0 | 0 | 0 | 1 | 67.88 | 37.10 | 30.78 |  |
| Control 6 | Control | F | 76 | Euthanasia | 08:35 | - | - | 3/3 | 1 | 2 | 0 | 0 | 0 | 0 | 1 | 2 | 68.87 | 41.03 | 27.84 |  |
| Control 7 | Control | M | 67 | Liver failure | 07:20 | - | - | 2/3 | 1 | 1 | 2 | 0 | 0 | 0 | 1 | 0 | 72.08 | 42.69 | 29.40 |  |
| Control 8 | Control | F | 72 | Heart failure | 08:00 | - | - | 3/3 | 0 | 0 | 0 | 0 | 0 | 0 | 0 | 1 | 77.37 | 45.40 | 31.97 |  |
| Control 9 | Control | F | 69 | Pulmonary embolism | 11:45 | - | - | 3/3 | 1 | 1 | 0 | 1 | 0 | 0 | 1 | 2 | 73.37 | 43.43 | 29.94 |  |
| Control 10 | Control | M | 59 | Euthanasia | 06:15 | - | - | 3/4 | 1 | 2 | 0 | 0 | 0 | 1 | 1 | 0 | 71.19 | 40.23 | 30.96 |  |
| Control 11 | Control | M | 77 | Pneumonia | 05:25 | - | - | 2/3 | 1 | 1 | 0 | 0 | 1 | 2 | 2 | 0 | 64.33 | 35.30 | 29.03 |  |
| Control 12 | Control | F | 79 | Unknown | 04:30 | - | - | NA | 2 | 3 | 0 | 0 | 0 | 0 | 0 | 0 | 72.67 | 42.08 | 30.60 |  |
| Control 13 | Control | F | 78 | Unknown | 08:10 | - | - | 3/3 | 1 | 1 | 0 | 1 | 0 | 0 | 0 | 2 | 68.44 | 38.50 | 29.94 |  |
| Control 14 | Control | F | 59 | Euthanasia | 10:20 | - | - | 3/3 | 0 | 0 | 0 | 0 | 0 | 1 | 0 | 2 | 67.52 | 38.67 | 28.85 |  |
| Control 15 | Control | F | 71 | Lung carcinoma | 08:35 | - | - | 3/4 | 1 | 2 | 1 | 0 | 1 | 1 | 0 | 2 | 71.34 | 40.85 | 30.49 |  |
| Control 16 | Control | M | 74 | Euthanasia | 09:15 | - | - | 3/3 | 2 | 3 | 0 | 0 | 1 | 1 | 1 | 1 | 60.70 | 35.99 | 24.71 |  |
| PMD is presented in hours:minutes, disease duration in years and NWBV, NGMV, and NWMV in percentage of estimated intracranial volume. APOE describes genotyping of present APOE isoforms.  M= male, F= female, PMD = post mortem delay, CDR = clinical dementia score, NFT = neurofibrillary tangles, LB = Lewy body, GCA = global cortical atrophy, PCA = parietal cortical atrophy, MTA = medial temporal lobe atrophy, NWBV = normalized whole brain volume, NGMV = normalized gray matter volume, NWMV = normalized white matter volume, NA= not available. | | | | | | | | | | | | | | | | | | | | |

**Supplementary Table 3. Cortical Volume and Pathological load per Phenotype group and subtypes.**

|  | **Region** | **Control** | **Typical AD** | **Atypical AD** | **Behavioural** | **Dysexecutive** | **Logopenic** | **Visuospatial** |
| --- | --- | --- | --- | --- | --- | --- | --- | --- |
| ***n*** |  | 16 | 17 | 16 | 3 | 5 | 3 | 4 |
| **Lobular volume** | Frontal | 100(±7.8) | 96.40(±14.32) | 91.63(±19.65) | 87.52(±23.65) | 97.8(±22.41) | 79.06(±18.28) | 92.9(±18.92) |
|  | Temporal | 100(±8.61) | 88.58(±13.82) | 86.37(±18.59) | 85.69(±18.46) | 91.67(±20.86) | 73.75(±21.71) | 86.3(±18.37) |
|  | Parietal | 100(±10.42) | 88.89(±13.77) | 88.16(±20.65) | 86.33(±22.62) | 91.24(±19.21) | 78.29(±25.95) | 89.36(±25.61) |
|  | Insular | 100(±12.57) | 85.69(±17.58) | 77.09(±27.74) | 76(±30.29) | 90.86(±32.6) | 56.19(±25.05) | 71.79(±22.31) |
|  | Limbic | 100(±9.13) | 92.43(±12.29) | 91.90(±15.66) | 87.28(±12.51) | 97.7(±21.35) | 80.27(±13.97) | 95.33(±11.97) |
|  | Occipital | 100(±6.82) | 97.95(±12.64) | 96.53(±18.08) | 93.94(±10.65) | 100.57(±9.92) | 88.88(±25.18) | 97.88(±30.02) |
| **Regional volume** | Hip | 100(±9.98) | 61.37(±25.3) | 65.85(±25.51) | 57.44(±28.9) | 74.46(±32.62) | 58.01(±5.79) | 59.86(±25.66) |
|  | ParaHip | 100(±8.75) | 86.70(±16.74) | 92.89(±12.28) | 91.53(±6.13) | 95.66(±19.29) | 87.3(±13.07) | 92.74(±7.32) |
|  | GFM | 100(±8.67) | 93.19(±14.74) | 94.24(±19.29) | 90.27(±19.73) | 104.33(±20.46) | 82.85(±21.06) | 88.62(±16.71) |
|  | GTM | 100(±8.69) | 89.61(±11.39) | 87.55(±15.53) | 88.32(±16.87) | 92.68(±17.50) | 78.52(±20) | 83.76(±11.34) |
|  | GPS | 100(±18) | 80.08(±20.57) | 82.91(±21.24) | 78.36(±15.01) | 88.17(±13.90) | 83.97(±30.6) | 68.52(±17.31) |
|  | Precun | 100(±11.86) | 92.84(±13.05) | 94.2(±16.1) | 96.21(±9.69) | 98.93(±15.84) | 88.60(±23.5) | 85.88(±15.04) |
|  | PCC | 100(±9.8) | 92.24(±15.3) | 99.11(±11.61) | 99.84(±9.53) | 99.41(±18.33) | 99.17(±12.37) | 96.5(±6.29) |
|  | OC | 100(±8.67) | 90.65(±14.31) | 92.05(±16.09) | 92.89(±15.05) | 100.08(±16.43) | 84.14(±18.12) | 83.63(±15.05) |
| **Amyloid** | Hip | 0.80(±0.51) | 1.49(±1.46) | 1.90(±1.61) | 1.04(±0.61) | 2.79(±2.25) | 0.66(±0.03) | 2.16(±1.45) |
|  | ParaHip | 1.16(±1.26) | 2.77(±2.36) | 3.91(±2.47) | 1.91(±0.66) | 4.49(±2.75) | 3.26(±0.45) | 5.28(±3.54) |
|  | GFM | 1.71(±2.54) | 4.41(±3.08) | 5.34(±3.45) | 3.96(±1.19) | 7.48(±4.87) | 3.27(±1.96) | 5.94(±2.78) |
|  | GTM | 1.36(±1.53) | 3.56(±2.23) | 4.51(±2.67) | 2.93(±1.90) | 5.09(±3.42) | 4.35(±3.05) | 5.19(±2.77) |
|  | GPS | 0.98(±1.12) | 3.99(±2.27) | 4.46(±2.7) | 2.09(±0.53) | 6.21(±3.29) | 4.19(±1.25) | 4.74(±2.95) |
|  | Precun | 1.87(±2.45) | 3.68(±3.07) | 4.47(±2.92) | 2.75(±1.06) | 4.47(±3.59) | 4.03(±0.41) | 6.16(±4.38) |
|  | PCC | 1.17(±0.93) | 3.82(±1.61) | 4.62(±2.43) | 3.23(±1.68) | 5.82(±3.08) | 3.85(±1.15) | 4.68(±3.04) |
|  | OC | 0.63(±0.65) | 2.41(±1.59) | 2.83(±1.83) | 1.38(±0.09) | 2.81(±2.11) | 3.19(±0.37) | 3.73(±2.73) |
| **pTau** | Hip | 1.09(±1.03) | 36.91(±12.25) | 39.54(±11.20) | 43.37(±5.26) | 43.37(±8.75) | 31.00(±7.77) | 35.84(±20.74) |
|  | ParaHip | 2.90(±4.40) | 45.40(±12.14) | 50.81(±12.11) | 54.87(±17.16) | 48.68(±10.33) | 54.93(±5.99) | 44.46(±15.23) |
|  | GFM | 0.10(±0.06) | 34.55(±26.05) | 35.82(±20.61) | 17.91(±15.49) | 44.69(±25.50) | 45.26(±15.99) | 33.71(±18.51) |
|  | GTM | 1.18(±3.41) | 39.91(±19.44) | 49.65(±19.46) | 34.89(±24.62) | 46.86(±24.50) | 52.74(±11.22) | 58.37(±14.04) |
|  | GPS | 0.16(±0.22) | 32.29(±20.72) | 44.35(±20.09) | 26.65(±23.40) | 46.93(±28.00) | 50.17(±9.24) | 48.93(±12.58) |
|  | Precun | 0.12(±0.10) | 27.39(±20.47) | 43.80(±22.18) | 31.14(±31.14) | 44.52(±26.91) | 36.97(±18.77) | 57.80(±11.98) |
|  | PCC | 0.19(±0.23) | 33.92(±19.79) | 47.30(±19.88) | 40.25(±34.69) | 49.66(±20.37) | 47.01(±18.63) | 48.08(±17.68) |
|  | OC | 0.13(±0.15) | 19.38(±17.68) | 14.24(±16.72) | 5.39(±5.93) | 6.87(±9.00) | 10.31(±12.72) | 36.47(±15.88) |
| **NfL** | Hip | 9.31(±1.93) | 11.07(±3.42) | 12.04(±2.84) | 11.79(±2.51) | 12.07(±3.46) | 13.41(±3.82) | 11.61(±2.67) |
|  | ParaHip | 6.31(±1.84) | 9.27(±3.18) | 9.58(±2.89) | 8.51(±2.83) | 10.61(±2.35) | 11.26(±4.3) | 7.43(±1.98) |
|  | GFM | 6.83(±2.39) | 9.67(±2.86) | 11.39(±2.89) | 10.06(±5.31) | 11.40(±1.57) | 13.64(±3.09) | 10.25(±1.94) |
|  | GTM | 6.70(±1.60) | 9.71(±3.85) | 11.66(±4.54) | 10.17(±2.22) | 10.07(±4.71) | 18.46(±1.28) | 11.19(±2.03) |
|  | GPS | 7.55(±3.40) | 10.89(±3.64) | 13.83(±3.83) | 13.58(±6.30) | 13.22(±3.27) | 16.78(±4.41) | 13.37(±2.58) |
|  | Precun | 9.19(±3.64) | 11.76(±4.64) | 14.19(±4.15) | 12.9(±5.21) | 13.25(±3.45) | 16.49(±6.39) | 15.91(±1.72) |
|  | PCC | 8.54(±1.90) | 10.85(±3.54) | 12.08(±3.83) | 11.32(±4.49) | 11.11(±2.93) | 16.76(±1.10) | 11.97(±3.10) |
|  | OC | 8.24(±2.38) | 11.26(±4.2) | 12.65(±4.84) | 10.64(±2.13) | 11.04(±4.37) | 18.84(±3.33) | 12.96(±4.83) |
| **COLIV** | Hip | 2.27(±0.56) | 2.58(±0.41) | 2.74(±0.65) | 3.02(±0.12) | 2.59(±0.77) | 3.27(±0.71) | 2.63(±0.24) |
|  | ParaHip | 2.39(±0.55) | 2.78(±0.39) | 3.23(±0.84) | 3.60(±0.19) | 2.79(±0.71) | 4.41(±0.45) | 2.92(±0.42) |
|  | GFM | 3.33(±0.62) | 3.73(±0.63) | 4.12(±1.08) | 4.32(±1.01) | 4.41(±1.52) | 4.73(±0.62) | 3.21(±0.43) |
|  | GTM | 2.54(±0.54) | 2.99(±0.56) | 3.66(±0.94) | 3.69(±0.35) | 3.14(±1.06) | 4.44(±1.06) | 3.94(±0.75) |
|  | GPS | 3.25(±0.57) | 3.98(±0.96) | 4.66(±0.97) | 4.10(±0.49) | 4.09(±0.57) | 5.74(±0.85) | 4.93(±1.21) |
|  | Precun | 3.34(±0.57) | 4.06(±1.04) | 4.33(±1.11) | 4.20(±0.33) | 3.90(±0.72) | 5.39(±1.78) | 4.70(±0.26) |
|  | PCC | 3.21(±0.76) | 4.15(±1.17) | 5.01(±1.57) | 4.23(±0.21) | 5.18(±2.29) | 6.34(±1.11) | 4.71(±1.18) |
|  | OC | 3.82(±0.60) | 4.34(±1.02) | 4.77(±1.22) | 4.99(±1.00) | 3.79(±0.28) | 6.14(±1.17) | 4.91(±1.45) |
| Data is presented as mean (± standard deviation). Hip = hippocampus, ParaHip = parahippocampal gyrus, GFM = middle frontal gyrus, GTM = middle temporal gyrus, GPS = superior parietal gyrus, Precun = precuneus, PCC = posterior cingulate cortex, OC = occipital cortex. | | | | | | | | |

**Supplementary table 4. Significant results from group comparisons in regional volume of AAL3 regions.**

| **AAL3 Region** | **Major divisions** | **Contrast** | **Estimate** | **EMM Group1** | **EMM Group2** | **T value** | **Effect Size** | **P value** | **Adjusted p** |
| --- | --- | --- | --- | --- | --- | --- | --- | --- | --- |
| IFGoperc_L | Frontal | Control - Typical AD | 4.774 | 32.825 | 28.051 | 3.613 | -1.353 | 0.002 | 0.017 |
| OLF_L | Frontal | Control - Atypical AD | 4.648 | 23.906 | 19.846 | 3.473 | -1.377 | 0.004 | 0.031 |
| OLF_L | Frontal | Control - Typical AD | 4.060 | 23.906 | 19.846 | 3.212 | -1.202 | 0.007 | 0.036 |
| PreCG_L | Frontal | Control - Typical AD | 1.728 | 15.227 | 13.499 | 3.082 | -1.154 | 0.010 | 0.046 |
| ANG_L | Parietal | Control - Typical AD | 53.262 | 372.022 | 318.760 | 3.503 | -1.312 | 0.003 | 0.021 |
| IPG_L | Parietal | Control - Typical AD | 107.300 | 651.299 | 543.999 | 3.147 | -1.178 | 0.009 | 0.039 |
| IPG_R | Parietal | Control - Typical AD | 63.542 | 294.883 | 231.340 | 3.030 | -1.134 | 0.012 | 0.048 |
| PoCG_L | Parietal | Control - Typical AD | 154.420 | 957.995 | 803.575 | 4.067 | -1.523 | 0.001 | 0.007 |
| PoCG_R | Parietal | Control - Typical AD | 118.061 | 763.229 | 645.167 | 3.250 | -1.217 | 0.006 | 0.035 |
| SMG_L | Parietal | Control - Typical AD | 82.990 | 406.106 | 323.117 | 4.412 | -1.652 | <0.001 | 0.003 |
| SMG_R | Parietal | Control - Typical AD | 95.076 | 542.165 | 447.090 | 3.949 | -1.478 | 0.001 | 0.009 |
| FFG_L | Temporal | Control - Atypical AD | 81.941 | 710.378 | 636.828 | 3.362 | -1.333 | 0.005 | 0.038 |
| FFG_L | Temporal | Control - Typical AD | 73.550 | 710.378 | 636.828 | 3.195 | -1.196 | 0.008 | 0.036 |
| HES_L | Temporal | Control - Atypical AD | 18.065 | 76.934 | 57.337 | 3.356 | -1.330 | 0.005 | 0.038 |
| HES_L | Temporal | Control - Typical AD | 19.597 | 76.934 | 57.337 | 3.854 | -1.443 | 0.001 | 0.011 |
| ITG_L | Temporal | Control - Atypical AD | 213.955 | 1481.983 | 1272.188 | 4.124 | -1.635 | 0.001 | 0.012 |
| ITG_L | Temporal | Control - Typical AD | 209.795 | 1481.983 | 1272.188 | 4.281 | -1.603 | <0.001 | 0.004 |
| ITG_R | Temporal | Control - Typical AD | 192.768 | 1327.228 | 1134.460 | 3.307 | -1.238 | 0.006 | 0.032 |
| MTG_L | Temporal | Control - Atypical AD | 332.465 | 2124.683 | 1822.658 | 4.642 | -1.840 | <0.001 | 0.004 |
| MTG_L | Temporal | Control - Typical AD | 302.026 | 2124.683 | 1822.658 | 4.465 | -1.672 | <0.001 | 0.003 |
| MTG_R | Temporal | Control - Atypical AD | 229.124 | 1612.663 | 1398.479 | 3.310 | -1.312 | 0.006 | 0.040 |
| MTG_R | Temporal | Control - Typical AD | 214.184 | 1612.663 | 1398.479 | 3.276 | -1.226 | 0.006 | 0.033 |
| STG_L | Temporal | Control - Atypical AD | 189.405 | 875.760 | 734.604 | 4.318 | -1.711 | <0.001 | 0.008 |
| STG_L | Temporal | Control - Typical AD | 141.157 | 875.760 | 734.604 | 3.407 | -1.275 | 0.004 | 0.026 |
| STG_R | Temporal | Control - Atypical AD | 171.682 | 1113.100 | 952.272 | 3.532 | -1.400 | 0.003 | 0.027 |
| STG_R | Temporal | Control - Typical AD | 160.828 | 1113.100 | 952.272 | 3.503 | -1.311 | 0.003 | 0.021 |
| TPOmid_L | Temporal | Control - Atypical AD | 52.193 | 253.958 | 204.835 | 3.618 | -1.434 | 0.002 | 0.027 |
| TPOmid_L | Temporal | Control - Typical AD | 49.123 | 253.958 | 204.835 | 3.605 | -1.350 | 0.002 | 0.017 |
| TPOsup_L | Temporal | Control - Typical AD | 62.928 | 246.017 | 183.089 | 3.626 | -1.358 | 0.002 | 0.017 |
| CAL_R | Occipital | Control - Typical AD | 66.862 | 496.616 | 429.754 | 3.202 | -1.199 | 0.007 | 0.036 |
| SOG_L | Occipital | Control - Typical AD | 35.920 | 306.422 | 270.502 | 3.032 | -1.135 | 0.012 | 0.048 |
| ACCpre_L | Limbic | Control - Atypical AD | 75.819 | 384.231 | 329.893 | 3.910 | -1.550 | 0.001 | 0.014 |
| ACCpre_R | Limbic | Control - Atypical AD | 68.449 | 522.761 | 479.294 | 3.200 | -1.268 | 0.007 | 0.046 |
| HIP_L | Limbic | Control - Atypical AD | 67.942 | 187.794 | 117.142 | 5.202 | -2.062 | <0.001 | 0.002 |
| HIP_L | Limbic | Control - Typical AD | 70.651 | 187.794 | 117.142 | 5.726 | -2.144 | <0.001 | <0.001 |
| HIP_R | Limbic | Control - Atypical AD | 73.671 | 201.509 | 120.032 | 4.728 | -1.874 | <0.001 | 0.003 |
| HIP_R | Limbic | Control - Typical AD | 81.477 | 201.509 | 120.032 | 5.535 | -2.072 | <0.001 | <0.001 |
| PCC_R | Limbic | Control - Typical AD | 8.927 | 79.530 | 70.603 | 3.072 | -1.150 | 0.010 | 0.046 |
| PHG_L | Limbic | Control - Atypical AD | 26.258 | 194.609 | 154.298 | 3.249 | -1.288 | 0.007 | 0.044 |
| PHG_L | Limbic | Control - Typical AD | 40.310 | 194.609 | 154.298 | 5.280 | -1.977 | <0.001 | <0.001 |
| PHG_R | Limbic | Control - Typical AD | 48.822 | 286.754 | 237.932 | 5.013 | -1.877 | <0.001 | 0.001 |
| AMYG_L | Subcortical | Control - Atypical AD | 6.662 | 51.746 | 42.501 | 3.321 | -1.316 | 0.005 | 0.040 |
| AMYG_L | Subcortical | Control - Typical AD | 9.245 | 51.746 | 42.501 | 4.879 | -1.827 | <0.001 | 0.001 |
| AMYG_R | Subcortical | Control - Typical AD | 7.634 | 62.902 | 55.268 | 4.482 | -1.678 | <0.001 | 0.003 |
| CAU_L | Subcortical | Control - Atypical AD | 79.926 | 148.643 | 68.638 | 4.102 | -1.626 | 0.001 | 0.012 |
| CAU_L | Subcortical | Control - Typical AD | 80.005 | 148.643 | 68.638 | 4.346 | -1.627 | <0.001 | 0.004 |
| CAU_R | Subcortical | Control - Typical AD | 124.252 | 286.667 | 162.415 | 4.006 | -1.500 | 0.001 | 0.008 |
| IFGtriang_L | Frontal | Control - Atypical AD | 13.126 | 96.982 | 86.995 | 3.224 | -1.278 | 0.007 | 0.044 |
| OFCpost_L | Frontal | Control - Atypical AD | 15.814 | 71.872 | 61.228 | 3.984 | -1.579 | 0.001 | 0.013 |
| tAV_L | Subcortical | Control - Atypical AD | 4.993 | 14.104 | 9.629 | 3.695 | -1.464 | 0.002 | 0.024 |
| tAV_L | Subcortical | Control - Typical AD | 4.475 | 14.104 | 9.629 | 3.506 | -1.312 | 0.003 | 0.021 |
| tAV_R | Subcortical | Control - Atypical AD | 6.235 | 15.810 | 9.798 | 3.577 | -1.418 | 0.003 | 0.027 |
| tAV_R | Subcortical | Control - Typical AD | 6.013 | 15.810 | 9.798 | 3.652 | -1.367 | 0.002 | 0.017 |
| tLP_L | Subcortical | Control - Atypical AD | 9.484 | 15.217 | 5.841 | 5.154 | -2.043 | <0.001 | 0.002 |
| tLP_L | Subcortical | Control - Typical AD | 9.376 | 15.217 | 5.841 | 5.395 | -2.020 | <0.001 | <0.001 |
| tLP_R | Subcortical | Control - Atypical AD | 8.798 | 14.369 | 5.251 | 4.816 | -1.909 | <0.001 | 0.003 |
| tLP_R | Subcortical | Control - Typical AD | 9.119 | 14.369 | 5.251 | 5.284 | -1.978 | <0.001 | <0.001 |
| tMDm_R | Subcortical | Control - Typical AD | 26.050 | 89.769 | 63.718 | 3.212 | -1.202 | 0.007 | 0.036 |
| tPuI_R / tPuL_R | Subcortical | Control - Atypical AD | 6.145 | 21.420 | 14.569 | 3.940 | -1.562 | 0.001 | 0.014 |
| tPuI_R / tPuL_R | Subcortical | Control - Typical AD | 6.851 | 21.420 | 14.569 | 4.650 | -1.741 | <0.001 | 0.002 |
| tPuM_L | Subcortical | Control - Atypical AD | 33.406 | 153.923 | 121.669 | 3.236 | -1.283 | 0.007 | 0.044 |
| tPuM_L | Subcortical | Control - Typical AD | 32.254 | 153.923 | 121.669 | 3.308 | -1.238 | 0.006 | 0.032 |
| tPuM_R | Subcortical | Control - Atypical AD | 34.885 | 135.203 | 99.449 | 3.538 | -1.402 | 0.003 | 0.027 |
| tPuM_R | Subcortical | Control - Typical AD | 35.754 | 135.203 | 99.449 | 3.839 | -1.437 | 0.001 | 0.011 |
| tVA_R | Subcortical | Control - Typical AD | 11.464 | 59.545 | 48.081 | 3.642 | -1.363 | 0.002 | 0.017 |
| tVL_L | Subcortical | Control - Atypical AD | 15.527 | 218.560 | 209.792 | 4.052 | -1.606 | 0.001 | 0.012 |
| Nacc_L / Vent_Str_L | Brainstem | Control - Atypical AD | 24.279 | 144.810 | 130.980 | 3.551 | -1.408 | 0.003 | 0.027 |
| AAL3 regions are abbreviated according to the AAL3 atlas specifications and corresponding to figure 2. EMM = Estimated Marginal Mean, retrieved in post-hoc fashion from linear regression model. Significance was set at p ≤ 0.05 after groupwise fdr multiple comparison correction. | | | | | | | | | |

**Supplementary Table 5. Correlation analysis between MRI volume measurements and visual atrophy scores.**

|  |  | **GCA** | |  | **MTA** | |  | **PCA** | |
| --- | --- | --- | --- | --- | --- | --- | --- | --- | --- |
|  |  | **r** | ***p*** |  | **r** | ***p*** |  | **r** | ***p*** |
| **All cases** |  |  |  |  |  |  |  |  |  |
|  | **NWBV** | **-0.794** | <.001***** | **Hip** | **-0.846** | <.001***** | **GPS** | **-0.519** | 0.001***** |
|  | **NGMV** | **-0.727** | <.001***** | **ParaHip** | **-0.585** | <.001***** | **Precun** | **-0.447** | 0.001***** |
|  | **NWMV** | **-0.641** | <.001***** |  |  |  | **Parietal lobe** | **-0.607** | <.001***** |
| **Control** |  |  |  |  |  |  |  |  |  |
|  | **NWBV** | -0.434 | 0.093 | **Hip** | -0.290 | 0.276 | **GPS** | -0.450 | 0.080 |
|  | **NGMV** | -0.469 | 0.067 | **ParaHip** | 0.057 | 0.834 | **Precun** | -0.274 | 0.304 |
|  | **NWMV** | -0.399 | 0.125 |  |  |  | **Parietal lobe** | -0.486 | 0.056 |
| **AD** |  |  |  |  |  |  |  |  |  |
|  | **NWBV** | **-0.707** | <.001***** | **Hip** | **-0.830** | <.001***** | **GPS** | **-0.404** | 0.020***** |
|  | **NGMV** | **-0.657** | <.001***** | **ParaHip** | **-0.617** | <.001***** | **Precun** | **-0.493** | 0.004***** |
|  | **NWMV** | **-0.409** | 0.018***** |  |  |  | **Parietal lobe** | **-0.603** | <.001***** |
| **Typical AD** |  |  |  |  |  |  |  |  |  |
|  | **NWBV** | **-0.587** | 0.013***** | **Hip** | **-0.779** | <.001***** | **GPS** | -0.207 | 0.425 |
|  | **NGMV** | -0.394 | 0.117 | **ParaHip** | **-0.723** | 0.001***** | **Precun** | -0.409 | 0.103 |
|  | **NWMV** | -0.422 | 0.092 |  |  |  | **Parietal lobe** | -0.437 | 0.079 |
| **Atypical AD** |  |  |  |  |  |  |  |  |  |
|  | **NWBV** | **-0.798** | <.001***** | **Hip** | **-0.788** | <.001***** | **GPS** | **-0.533** | 0.034***** |
|  | **NGMV** | **-0.782** | <.001***** | **ParaHip** | **-0.513** | 0.041***** | **Precun** | **-0.653** | 0.006***** |
|  | **NWMV** | -0.468 | 0.067 |  |  |  | **Parietal lobe** | **-0.768** | <.001***** |
|  | GCA = global cortical atrophy, PCA = parietal cortical atrophy, MTA = medial temporal lobe atrophy, NWBV = normalized whole brain volume, NGMV = normalized gray matter volume, NWMV = Normalized white matter volume. Hip = hippocampus medius, ParaHip = parahippocampal gyrus, GPS = gyrus parietalis superior, Precun = precuneus. Significant correlation coefficients are presented in bold and significant p-values are marked with *****. | | | | | | | | |

**Supplementary Table 6. Correlation analysis between pathologies**

|  | **Amyloid** | | **pTau** | | **NFL** | | **COLIV** | |
| --- | --- | --- | --- | --- | --- | --- | --- | --- |
|  | **r** | **p** | **r** | **p** | **r** | **p** | **r** | **p** |
| **All cases** |  |  |  |  |  |  |  |  |
| pTau | **0.507** | <.001***** | - | - |  |  |  |  |
| NfL | **0.199** | <.001***** | **0.451** | <.001***** | - | - |  |  |
| COLIV | **0.184** | <.001***** | **0.233** | <.001***** | **0.457** | <.001***** | - | - |
| Ratio | **-0.163** | 0.004***** | -0.084 | 0.382 | -0.024 | 0.636 | -0.061 | 0.257 |
| **Control** |  |  |  |  |  |  |  |  |
| pTau | 0.088 | 0.518 | - | - |  |  |  |  |
| NfL | -0.048 | 0.740 | 0.137 | 0.336 | - | - |  |  |
| COLIV | -0.204 | 0.262 | -0.152 | 0.336 | 0.157 | 0.336 | - | - |
| Ratio | -0.012 | 0.893 | -0.041 | 0.741 | 0.109 | 0.482 | 0.084 | 0.518 |
| **AD cases** |  |  |  |  |  |  |  |  |
| pTau | **0.281** | <.001***** | - | - |  |  |  |  |
| NFL | -0.059 | 0.562 | **0.233** | <.001***** | - | - |  |  |
| COLIV | -0.053 | 0.580 | 0.041 | 0.625 | **0.430** | <.001***** | - | - |
| Ratio | **-0.145** | 0.050***** | 0.040 | 1 | 0.066 | 0.562 | -0.036 | 0.625 |
| **Typical AD cases** |  |  |  |  |  |  |  |  |
| pTau | **0.260** | 0.009***** | - | - |  |  |  |  |
| NfL | -0.121 | 0.319 | 0.211 | 0.056 | - | - |  |  |
| COLIV | <.001 | 0.993 | -0.096 | 0.464 | **0.338** | <.001***** | - | - |
| Ratio | -0.014 | 0.993 | -0.011 | 0.993 | -0.061 | 0.698 | -0.189 | 0.103 |
| **Atypical AD cases** |  |  |  |  |  |  |  |  |
| pTau | **0.287** | 0.004***** | - | - |  |  |  |  |
| NfL | -0.066 | 0.512 | 0.204 | 0.083 | - | - |  |  |
| COLIV | 0.072 | 0.512 | 0.095 | 0.411 | **0.500** | <.001***** | - | - |
| Ratio | **-0.290** | 0.005***** | 0.003 | 0.967 | 0.184 | 0.075 | 0.099 | 0.411 |
| Ratio is defined as microvascular wall thickness/microvascular area. Significant correlation coefficients are presented in bold and significant p-values are marked with *****. | | | | | | | | |

**Supplementary Table 7. Correlation analysis between CAA and Aβ load, COLIV load and ratio.**

|  | **CAA severity score** | |
| --- | --- | --- |
|  | **r** | ***p*** |
| **All cases** |  |  |
| Amyloid | **0.335** | <.001* |
| COLIV | **0.311** | <.001* |
| Ratio | **-0.401** | <.001* |
| **Control** |  |  |
| Amyloid | **0.181** | 0.041* |
| COLIV | -0.069 | 0.457 |
| Ratio | -0.016 | 1 |
| **AD cases** |  |  |
| Amyloid | 0.0869 | 0.138 |
| COLIV | **0.2034** | 0.004* |
| Ratio | **-0.4526** | <.001* |
| **Typical AD cases** |  |  |
| Amyloid | -0.050 | 0.512 |
| COLIV | **0.440** | <.001* |
| Ratio | **-0.536** | <.001* |
| **Atypical AD cases** |  |  |
| Amyloid | **0.353** | <.001* |
| COLIV | -0.048 | 0.5943 |
| Ratio | **-0.353** | <.001* |
| Significant correlation coefficients are presented in bold and significant p-values are marked with *****. | | |

**Supplementary Table 8. Linear mixed model results regional volume pathology associations.**

|  | **Controls** | | | | **Typical AD** | | | | **Atypical AD** | | | |
| --- | --- | --- | --- | --- | --- | --- | --- | --- | --- | --- | --- | --- |
|  | **β** | **βs** | **t ratio** | ***p*** | **β** | **βs** | **t ratio** | ***p*** | **β** | **βs** | **t ratio** | ***p*** |
| **Amyloid** |  |  |  |  |  |  |  |  |  |  |  |  |
| Hip | 2.809 (5.283) | 0.395 (0.743) | 0.532 | 0.595 | **5.109 (1.812)** | **0.718 (0.255)** | 2.819 | 0.005* | **5.107 (1.755)** | **0.718 (0.247)** | 2.911 | 0.004* |
| ParaHip | -0.403 (2.188) | -0.057 (0.308) | -0.184 | 0.854 | 0.183 (1.117) | 0.026 (0.157) | 0.164 | 0.870 | 1.108 (1.158) | 0.156 (0.163) | 0.956 | 0.340 |
| GFM | 0.553 (1.030) | 0.078 (0.145) | 0.536 | 0.592 | 0.305 (0.855) | 0.043 (0.120) | 0.357 | 0.721 | 0.605 (0.822) | 0.085 (0.116) | 0.735 | 0.463 |
| GTM | -0.015 (1.755) | -0.002 (0.247) | -0.008 | 0.993 | -0.541 (1.185) | -0.076 (0.167) | -0.456 | 0.649 | 0.370 (1.057) | 0.052 (0.149) | 0.350 | 0.727 |
| GPS | -2.170 (2.393) | -0.305 (0.336) | -0.907 | 0.365 | **3.353 (1.148)** | **0.471 (0.161)** | 2.920 | 0.004* | -0.457 (1.054) | -0.064 (0.148) | -0.434 | 0.665 |
| Precun | 0.736 (1.109) | 0.104 (0.156) | 0.664 | 0.507 | 0.090 (0.837) | 0.013 (0.118) | 0.108 | 0.914 | -0.503 (1.000) | -0.071 (0.141) | -0.503 | 0.615 |
| PCC | -0.182 (2.922) | -0.026 (0.411) | -0.062 | 0.950 | -1.180 (1.625) | -0.166 (0.229) | -0.726 | 0.468 | 0.199 (1.167) | 0.028 (0.164) | 0.171 | 0.865 |
| OC | 1.584 (4.173) | 0.223 (0.587) | 0.379 | 0.705 | -1.544 (1.634) | -0.217 (0.230) | -0.945 | 0.345 | 1.820 (1.513) | 0.256 (0.213) | 1.203 | 0.230 |
| **pTau** |  |  |  |  |  |  |  |  |  |  |  |  |
| Hip | - | - | - | - | -4.517 (5.080) | -0.284 (0.320) | -0.889 | 0.375 | -3.824 (4.020) | 0.241 (0.276) | -0.951 | 0.343 |
| ParaHip | - | - | - | - | -2.855 (5.660) | -0.180 (0.356) | -0.505 | 0.614 | -0.719 (3.140) | 0.248 (0.266) | -0.229 | 0.819 |
| GFM | - | - | - | - | 1.143 (4.220) | 0.072 (0.265) | 0.271 | 0.787 | -1.718 (4.020) | 0.038 (0.127) | -0.428 | 0.669 |
| GTM | - | - | - | - | 3.909 (4.890) | 0.246 (0.308) | 0.799 | 0.425 | -2.895 (2.830) | -0.164 (0.166) | -1.021 | 0.308 |
| GPS | - | - | - | - | 5.670 (4.780) | 0.356 (0.301) | 1.186 | 0.237 | -2.249 (2.640) | -0.039 (0.155) | -0.852 | 0.395 |
| Precun | - | - | - | - | 3.453 (4.760) | 0.217 (0.299) | 0.726 | 0.469 | -1.880 (2.450) | -0.127 (0.160) | -0.767 | 0.444 |
| PCC | - | - | - | - | 1.561 (3.470) | 0.098 (0.218) | 0.450 | 0.653 | -0.793 (2.170) | -0.087 (0.165) | -0.365 | 0.715 |
| OC | - | - | - | - | 0.427 (4.510) | 0.027 (0.283) | 0.095 | 0.925 | -3.294 (2.510) | -0.011 (0.181) | -1.312 | 0.191 |
| **NfL** |  |  |  |  |  |  |  |  |  |  |  |  |
| Hip | 0.395 (1.346) | 0.084 (0.288) | 0.293 | 0.770 | **-2.739 (0.740)** | **-0.585 (0.158)** | -3.700 | >.001* | **-2.688 (0.921)** | **-0.574 (0.197)** | -2.918 | 0.004* |
| ParaHip | -0.444 (1.466) | -0.095 (0.313) | -0.303 | 0.762 | -0.605 (0.802) | -0.129 (0.171) | -0.755 | 0.451 | -0.604 (0.903) | -0.129 (0.193) | -0.669 | 0.504 |
| GFM | -0.347 (1.083) | -0.074 (0.231) | -0.321 | 0.749 | -0.796 (0.890) | -0.170 (0.190) | -0.895 | 0.372 | **-2.319 (0.908)** | **-0.496 (0.194)** | -2.554 | 0.011* |
| GTM | -0.262 (1.680) | -0.056 (0.359) | -0.156 | 0.876 | -0.221 (0.661) | -0.047 (0.141) | -0.334 | 0.739 | -0.643 (0.588) | -0.137 (0.126) | -1.094 | 0.275 |
| GPS | -0.689 (0.766) | -0.147 (0.164) | -0.899 | 0.369 | -0.165 (0.697) | -0.035 (0.149) | -0.237 | 0.813 | -0.422 (0.694) | -0.091 (0.148) | -0.608 | 0.544 |
| Precun | -0.010 (0.720) | -0.002 (0.154) | -0.014 | 0.989 | -0.061 (0.551) | -0.013 (0.118) | -0.111 | 0.912 | -0.321 (0.639) | -0.069 (0.136) | -0.503 | 0.615 |
| PCC | -0.495 (1.413) | -0.106 (0.302) | -0.350 | 0.726 | 0.545 (0.713) | 0.116 (0.152) | 0.764 | 0.445 | 0.758 (0.690) | 0.162 (0.148) | 1.097 | 0.273 |
| OC | 0.151 (1.102) | 0.032 (0.235) | 0.137 | 0.891 | 0.154 (0.606) | 0.033 (0.129) | 0.255 | 0.799 | -0.128 (0.550) | -0.027 (0.118) | -0.232 | 0.817 |
| **COLIV** |  |  |  |  |  |  |  |  |  |  |  |  |
| Hip | -4.517 (5.080) | -0.284 (0.320) | -0.889 | 0.375 | **-26.902 (6.670)** | **-1.691 (0.420)** | -4.030 | >.001* | -3.824 (4.020) | -0.240 (0.253) | -0.951 | 0.343 |
| ParaHip | -2.855 (5.660) | -0.180 (0.356) | -0.505 | 0.614 | -11.514 (6.880) | -0.724 (0.433) | -1.674 | 0.095 | -0.719 (3.140) | -0.045 (0.198) | -0.229 | 0.819 |
| GFM | 1.143 (4.220) | 0.072 (0.265) | 0.271 | 0.787 | -1.718 (4.02) | -0.108 (0.253) | -0.428 | 0.669 | -3.218 (2.41) | -0.202 (0.152) | -1.334 | 0.183 |
| GTM | 3.909 (4.890) | 0.246 (0.308) | 0.799 | 0.425 | 2.359 (4.510) | 0.148 (0.284) | 0.523 | 0.602 | -2.895 (2.830) | -0.182 (0.178) | -1.021 | 0.308 |
| GPS | 5.670 (4.780) | 0.357 (0.301) | 1.186 | 0.237 | -2.249 (2.640) | -0.141 (0.166) | -0.852 | 0.395 | -1.614 (2.73) | -0.102 (0.172) | -0.591 | 0.555 |
| Precun | 3.453 (4.760) | 0.217 (0.299) | 0.726 | 0.469 | -1.880 (2.450) | -0.118 (0.154) | -0.767 | 0.444 | -1.375 (2.400) | -0.087 (0.151) | -0.573 | 0.567 |
| PCC | 1.561 (3.470) | 0.098 (0.218) | 0.450 | 0.653 | -0.793 (2.170) | -0.050 (0.137) | -0.365 | 0.715 | 0.524 (1.690) | 0.033 (0.106) | 0.311 | 0.756 |
| OC | 0.427 (4.510) | 0.027 (0.283) | 0.095 | 0.925 | -3.294 (2.510) | -0.207 (0.158) | -1.312 | 0.191 | -0.629 (2.160) | -0.040 (0.136) | -0.291 | 0.771 |
| Data are presented β (± SE) for volume change rates. Estimates are based on raw values/scores and were estimated with linear mixed models. The models included the terms age, sex, post-mortem delay and estimated intracranial volume. Random slopes were assigned to subjects to account for the nested structure of the data and adjust for within subject correlations. Interaction term for clinical phenotype groups were added in order to study the contrast between groups. Separate models for each staining were used. Significant p-values are present in bold and marked with ***.** Hip = hippocampus, ParaHip = parahippocampal gyrus, GFM = middle frontal gyrus, GTM = middle temporal gyrus, GPS = superior parietal gyrus, Precun = precuneus, PCC = posterior cingulate cortex, OC = occipital cortex. | | | | | | | | | | | | |
