# Supplementary figures and images for: Clinical phenotypes of Alzheimer’s disease: investigating atrophy patterns and their pathological correlates
Source: Alzheimers Res Ther. 2025 Apr 26;17:93. doi: 10.1186/s13195-025-01727-5 (PMC12032798; doi:10.1186/s13195-025-01727-5)

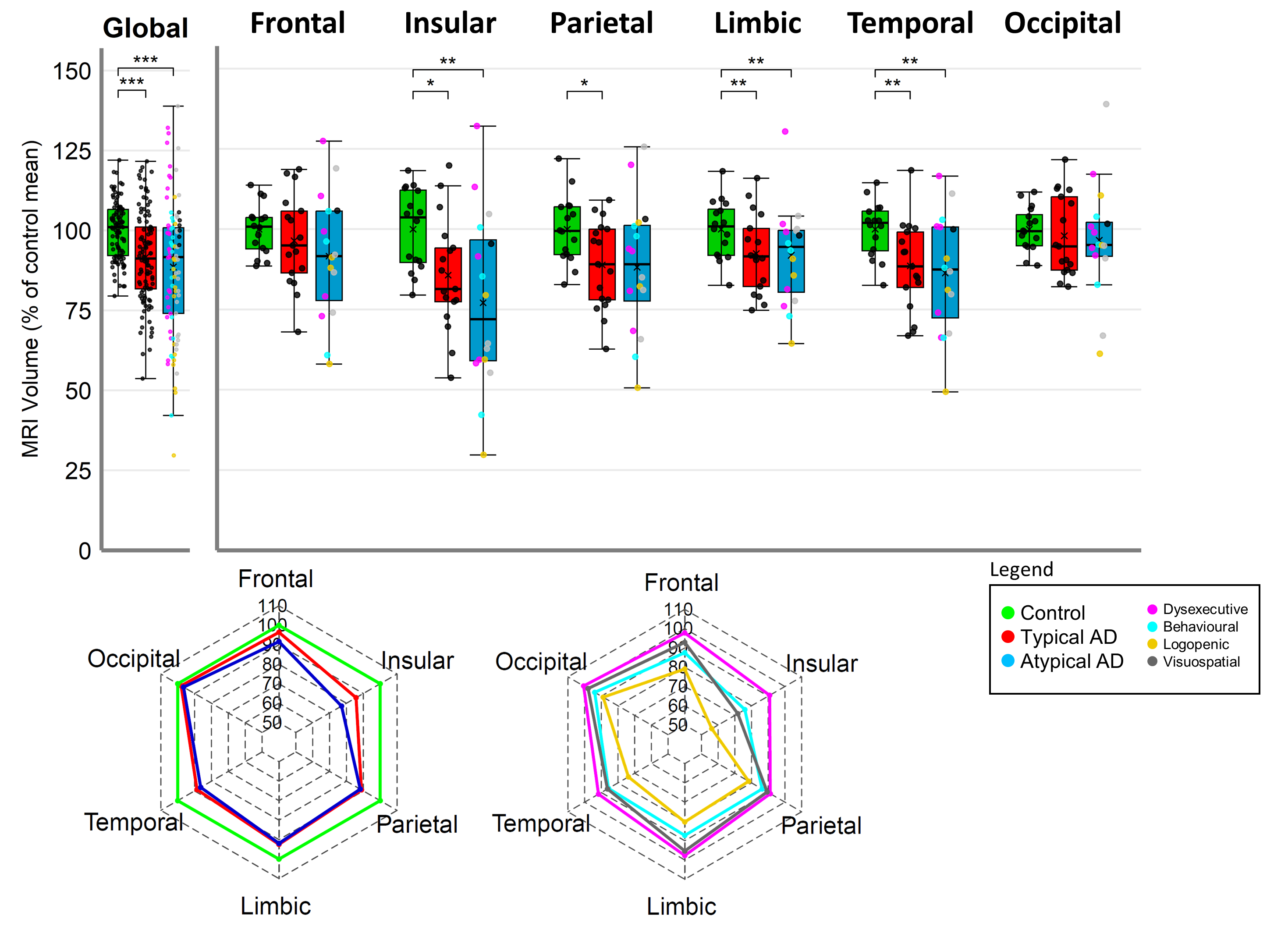

Supplement: Supplementary file 2 — Supplementary Material 2. Supplementary Figure 1. MRI lobular volume results. A) boxplots of lobular volumes across all cortical lobes of each group. Volumes are presented in boxplots as % of control mean volume which was set at 100% for each region. B) Radar plots of both clinical phenotypes and atypical subtypes, denoting the mean volume for each region per group. *= p ≤ 0.05, ** =p ≤ 0.01, *** =p ≤ 0.001. [file 13195_2025_1727_MOESM2_ESM.png]

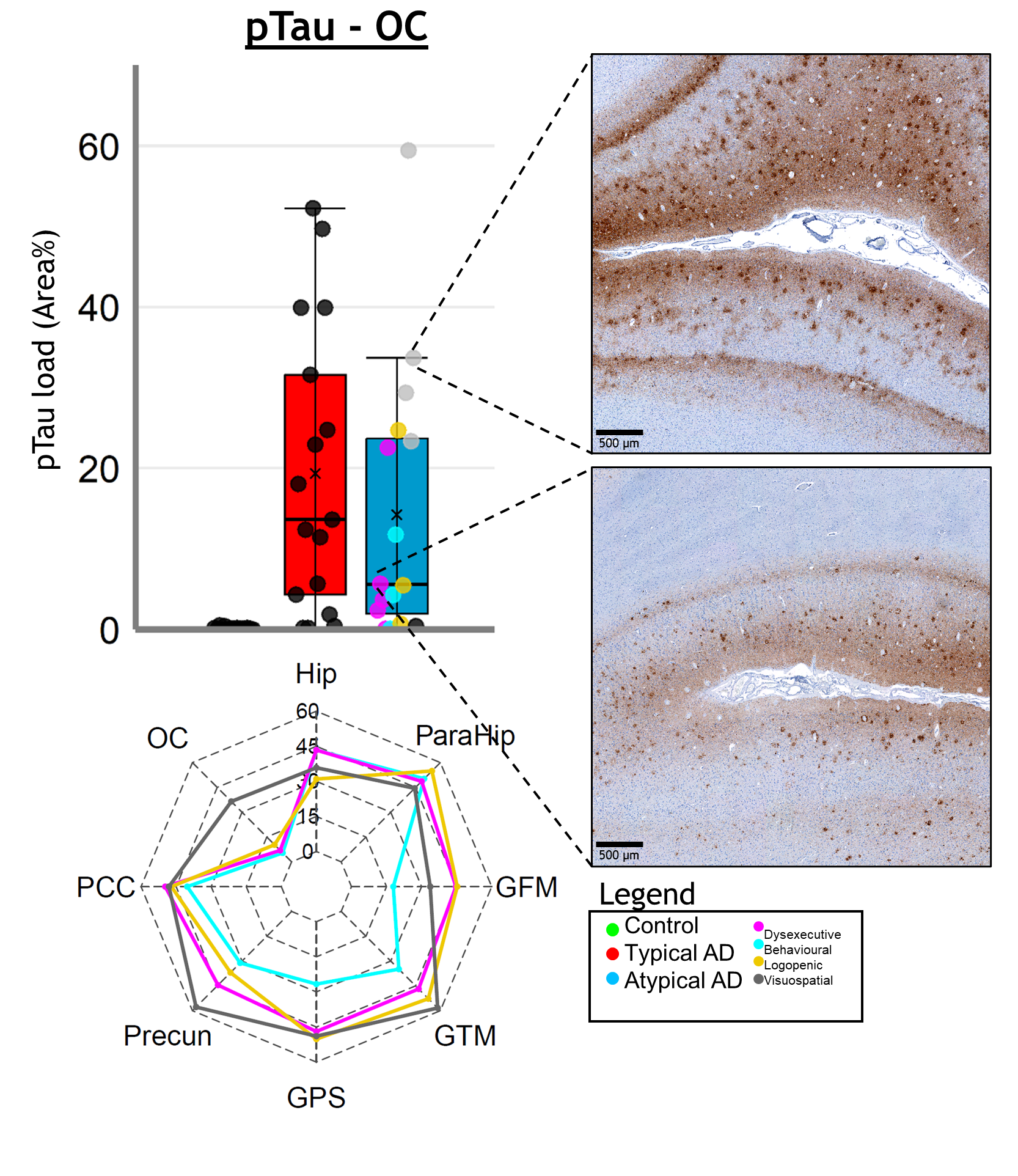

Supplement: Supplementary file 3 — Supplementary Material 3. Supplementary Figure 2. pTau load in the occipital cortex. A) boxplot of pTau load results of the occipital cortex of each group, with examples of high and low immunopositive staining for pTau. B) Radar plot atypical subtypes, denoting the mean volume for each region per group. Note the distinctly higher pTau load in the occipital cortex for the visuospatial group (grey). Hip = hippocampus, ParaHip = parahippocampal gyrus, GFM = middle frontal gyrus, GTM = middle temporal gyrus, GPS = superior parietal gyrus, Precun = precuneus, PCC = posterior cingulate cortex, OC = occipital cortex. [file 13195_2025_1727_MOESM3_ESM.png]

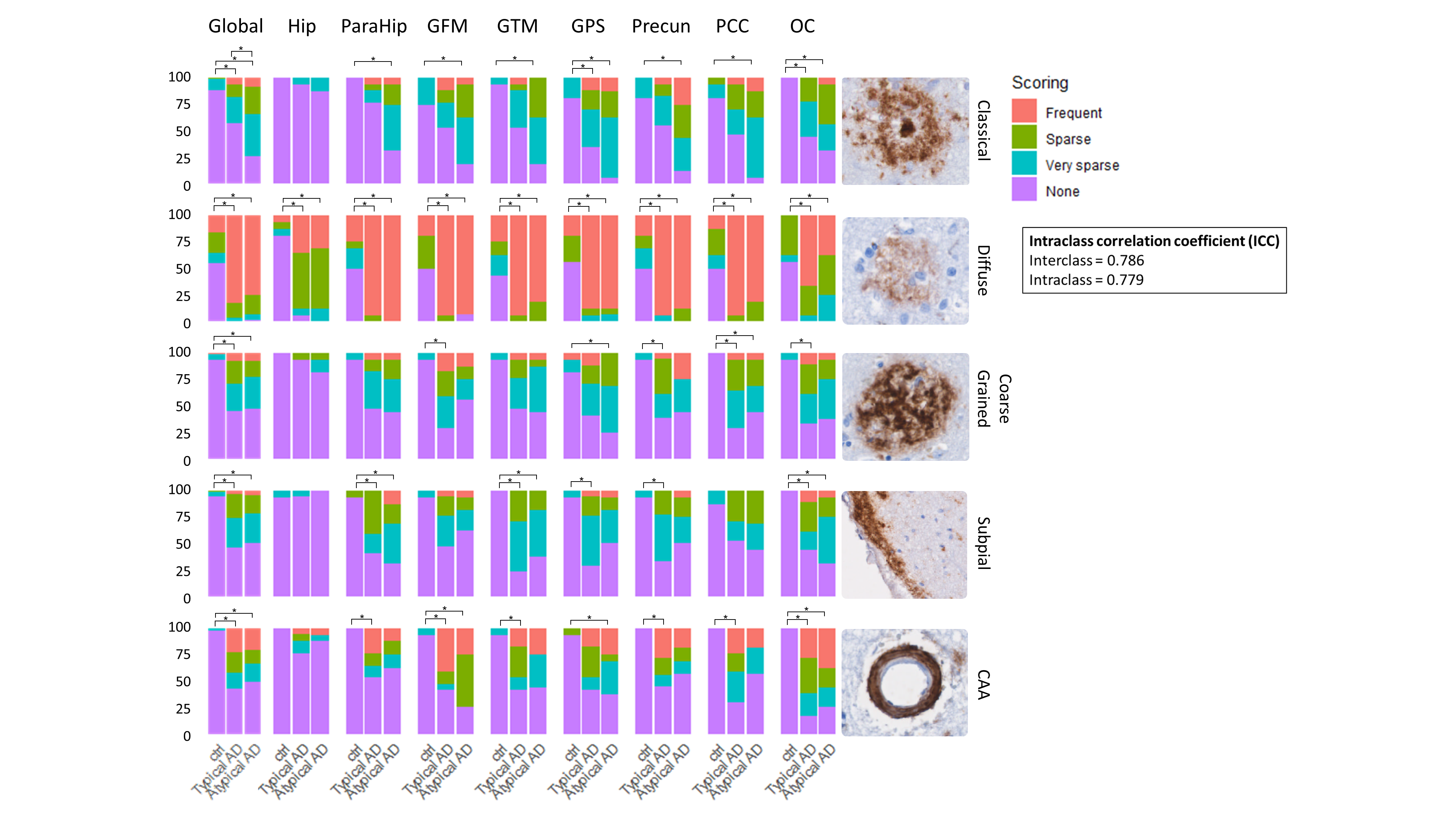

Supplement: Supplementary file 4 — Supplementary Material 4. Supplementary Figure 3. Semi-quantitative amyloid plaque scoring. Scoring (None, very sparse, sparse and frequent) as percentage of scoring observed in a group (control, typical AD or Atypical AD), totaling to 100%. * = p ≤ 0.05. Hip = hippocampus, ParaHip = parahippocampal gyrus, GFM = middle frontal gyrus, GTM = middle temporal gyrus, GPS = superior parietal gyrus, Precun = precuneus, PCC = posterior cingulate cortex, OC = occipital cortex [file 13195_2025_1727_MOESM4_ESM.png]

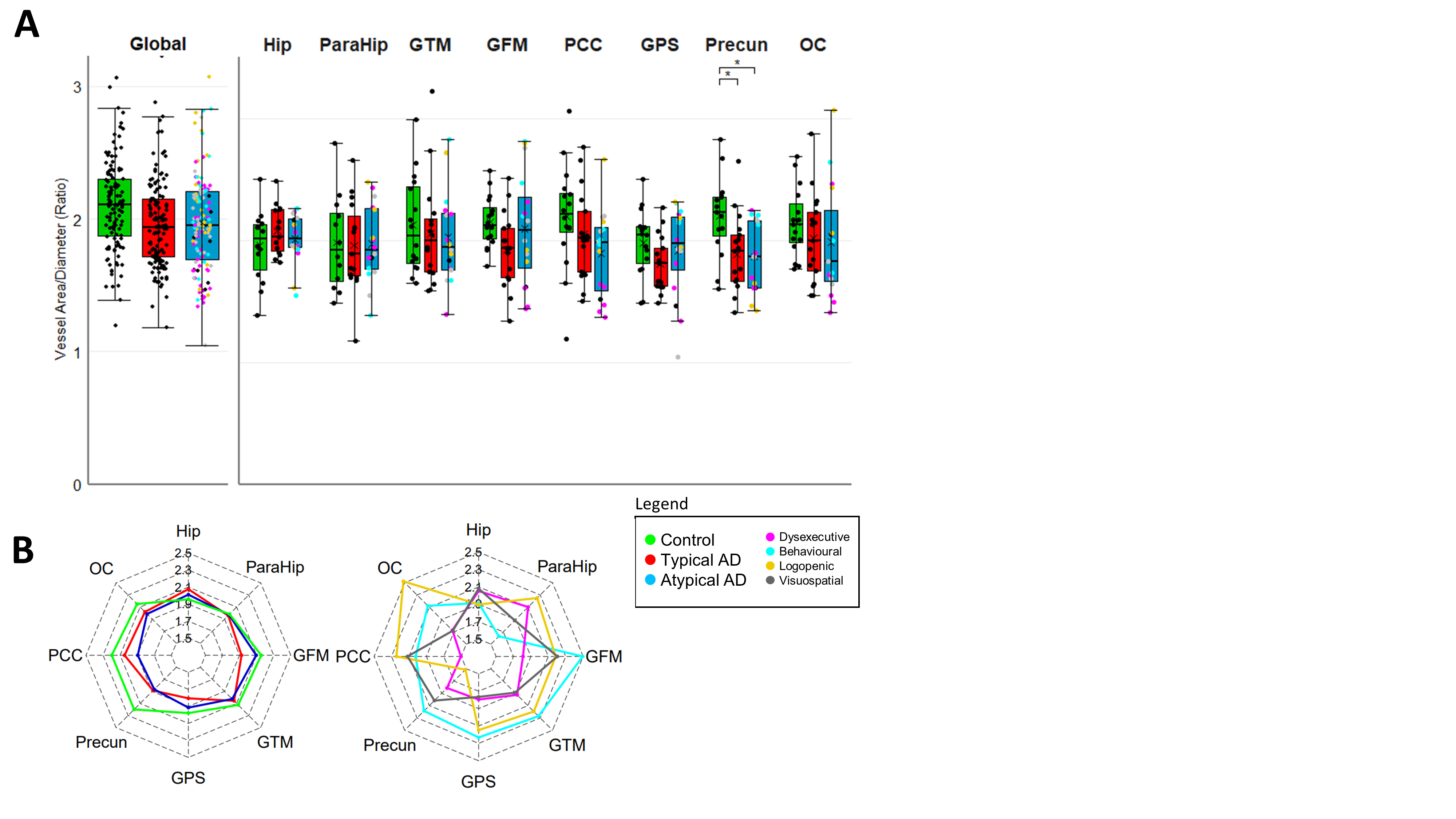

Supplement: Supplementary file 5 — Supplementary Material 5. Supplementary Figure 4. Microvascular vessel area : vessel diameter (ratio) quantification. A) Boxplots of the ratio across all cortical regions of each group, with only the precuneus showing significant differences between control and AD phenotype groups. B) Radarplots of both clinical phenotypes and atypical subtypes, denoting the mean ratio for each region per group. * = p ≤ 0.05.Hip = hippocampus, ParaHip = parahippocampal gyrus, GFM = middle frontal gyrus, GTM = middle temporal gyrus, GPS = superior parietal gyrus, Precun = precuneus, PCC = posterior cingulate cortex, OC = occipital cortex. [file 13195_2025_1727_MOESM5_ESM.png]

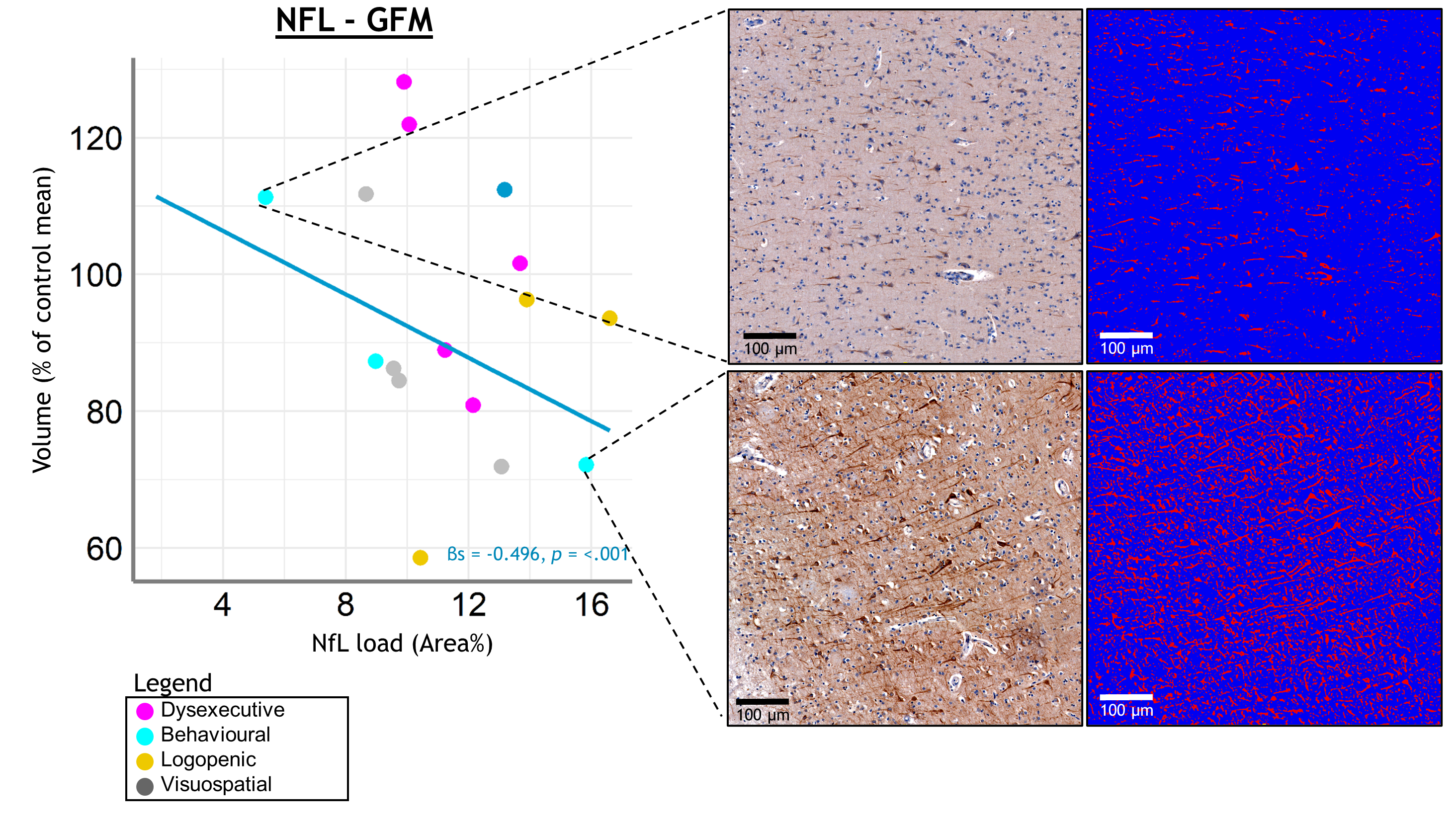

Supplement: Supplementary file 6 — Supplementary Material 6. Supplementary Figure 5. Association between volume and NfL in the middle frontal gyrus. Scatterplot with the linear mixed model derived regression line of hippocampal volume association with COLIV load for the atypical AD group, with its subtypes color coded. Examples of NFL immunoreactivity in the middle frontal gyrus are displayed of relatively low (top) and high (bottom) COLIV load both for the original image (left) and positive signal mask (right). [file 13195_2025_1727_MOESM6_ESM.png]

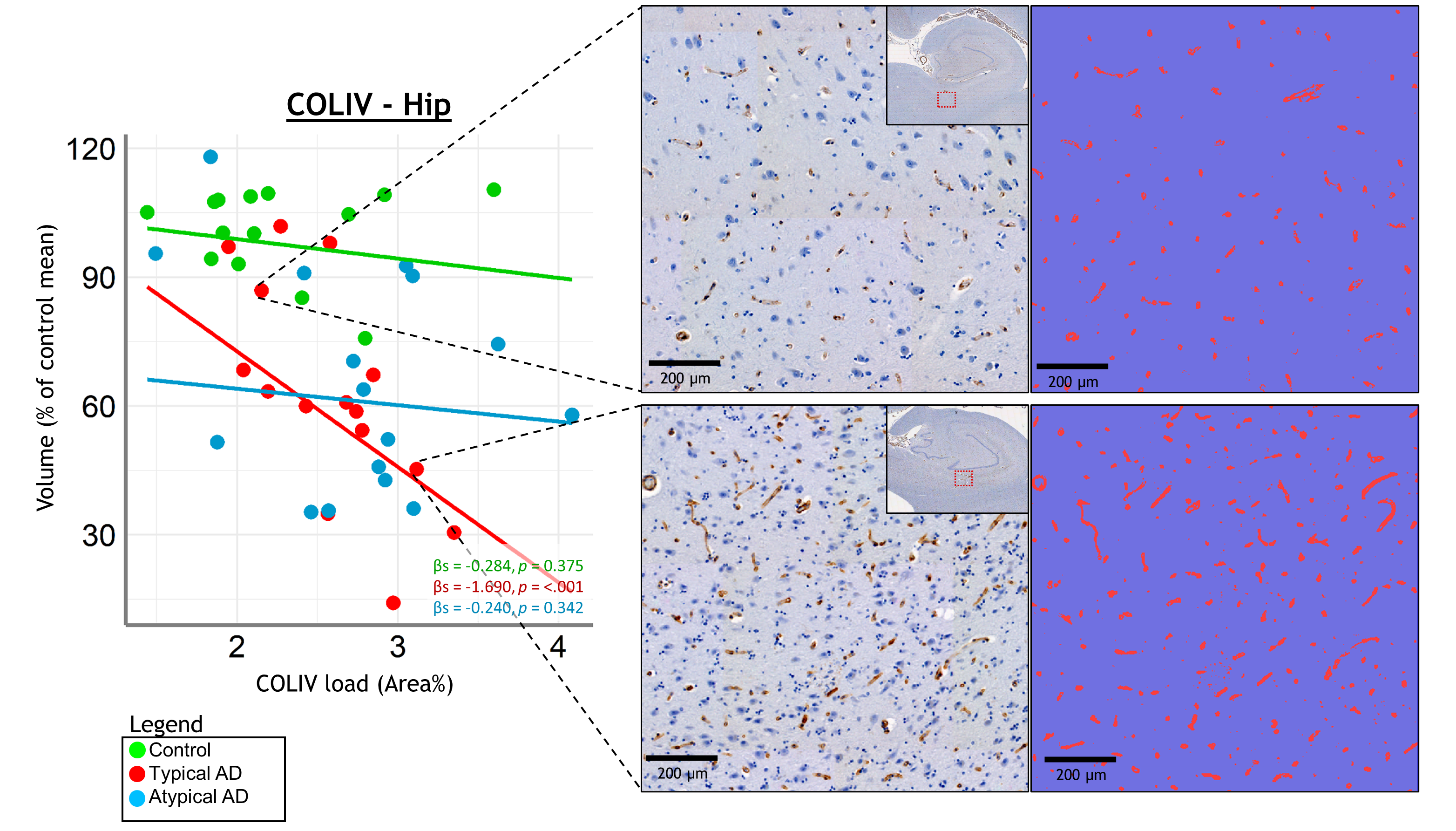

Supplement: Supplementary file 7 — Supplementary Material 7. Supplementary Figure 6. Association between hippocampal volume and Collagen IV. Scatterplot with the linear mixed model derived regression lines of hippocampal volume associations with COLIV load for each group. Examples of collagen IV immunoreactivity in the hippocampus (specifically in CA4/Subiculum crossover area) in typical AD cases are displayed of relatively low (top) and high (bottom) COLIV load for the original image (left) and positive signal mask (right). [file 13195_2025_1727_MOESM7_ESM.png]
